# Direct and Indirect Chemiluminescence: Reactions, Mechanisms and Challenges

**DOI:** 10.3390/molecules26247664

**Published:** 2021-12-17

**Authors:** Marina A. Tzani, Dimitra K. Gioftsidou, Michael G. Kallitsakis, Nikolaos V. Pliatsios, Natasa P. Kalogiouri, Panagiotis A. Angaridis, Ioannis N. Lykakis, Michael A. Terzidis

**Affiliations:** 1 Department of Chemistry, Aristotle University of Thessaloniki, University Campus, 54124 Thessaloniki, Greece; marina_tzani@hotmail.com (M.A.Tz.); dimgpan10@yahoo.gr (D.K.G.); kallitsos29@gmail.com (M.G.K.); npliatsios@gmail.com (N.V.P.); kalogiourin@gmail.com (N.P.K.); panosangaridis@chem.auth.gr (P.A.A.); 2 Department of Nutritional Sciences and Dietetics, International Hellenic University, Sindos Campus, 57400 Thessaloniki, Greece

**Keywords:** chemiluminescence, reaction mechanisms, singlet oxygen, reactive oxygen species, light emission

## Abstract

Emission of light by matter can occur through a variety of mechanisms. When it results from an electronically excited state of a species produced by a chemical reaction, it is called chemiluminescence (CL). The phenomenon can take place both in natural and artificial chemical systems and it has been utilized in a variety of applications. In this review, we aim to revisit some of the latest CL applications based on direct and indirect production modes. The characteristics of the chemical reactions and the underpinning CL mechanisms are thoroughly discussed in view of studies from the very recent bibliography. Different methodologies aiming at higher CL efficiencies are summarized and presented in detail, including CL type and scaffolds used in each study. The CL role in the development of efficient therapeutic platforms is also discussed in relation to the Reactive Oxygen Species (ROS) and singlet oxygen (^1^O_2_) produced, as final products. Moreover, recent research results from our team are included regarding the behavior of commonly used photosensitizers upon chemical activation under CL conditions. The CL prospects in imaging, biomimetic organic and radical chemistry, and therapeutics are critically presented in respect to the persisting challenges and limitations of the existing strategies to date.

## 1. Introduction

**Figure 1:**
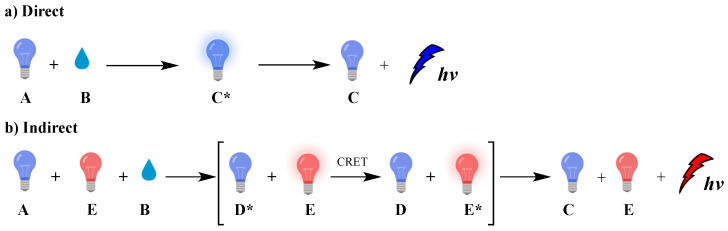
Direct (**a**) and indirect (**b**) chemiluminescence.

Chemiluminescence (CL) is the spontaneous emission of light from an electronically excited state of a species produced by a chemical reaction [1]. Nature uses CL (called bioluminescence) in many living organisms such as fireflies, mushrooms, shells, jellyfish, [2] worms and bacteria, mainly for communication or defense roles [3]. In biological systems, bioluminescence occurs from *in situ* enzyme-catalyzed chemical transformations, for example luciferin [4] reacts with oxygen in the presence of luciferase enzyme [5], magnesium [6,7,8,9] or calcium ions and adenosine triphosphate (ATP), leading to luminescence [10,11,12]. The reaction involves chemical activation of specific molecules (A) via oxidation, resulting in a chemiexcited intermediate (CEI) (C* or D*; excited species indicated by *) that releases its energy either via light emission (direct) or by transferring it, through a chemiluminescence resonance energy transfer (CRET) process, to an adjacent fluorophore (E) that becomes excited (E*); this fluorophore subsequently releases part of its energy by emitting light. These two distinct mechanisms dictate the two types of CL, direct and indirect, respectively, as shown in Figure 1.

Typical examples of molecules that emit light via direct CL are luminol and luciferin, two of the most well-studied luminogenic substances which are susceptible to oxidation by hydrogen peroxide (H_2_O_2_) and superoxide radical anion (O_2_^●–^). The emitted light is the result of changes in the chemical structure of the produced chemiexcited intermediates. Under certain conditions, singlet oxygen (^1^O_2_) can also be produced at high amounts, as chemiexcitation product, by peroxides via direct CL. ^1^O_2_ upon radiative relaxation emits infrared light at 1270 nm if not previously trapped, affording oxidation products [13]. The production of ^1^O_2_ under CL conditions is discussed later in the text (*vide infra*). In addition, oxalate esters and 1,2-dioxetane derivates serve as efficient precursors both for direct and indirect CL providing tunable emission systems depending on the photophysical properties of the energy acceptor fluorophore, with a wide range of applications in sensing and diagnosis, molecular imaging, and cancer treatment [14,15].

In indirect CL, energy transfer takes place, resulting in excitation of a fluorophore or a photosensitizer that can further act independently. In the presence of molecular oxygen (O_2_), the photosensitizer can generate reactive oxygen species (ROS) such as O_2_^●–^, hydroxyl radicals (HO^●^) or ^1^O_2_. These species have a broad spectrum of reactivity with biomolecules [16,17,18,19] and can be used in photodynamic therapy (PDT) to induce selectively cancer cell death, as well as other chemical transformations [20,21,22,23].

Most of the studied CL systems exhibit flash-type single-color light emissions that usually limit their applications. Long-lasting multicolor CL in aqueous solutions is highly desirable, especially in biological applications, however a controllable chemical procedure still remains a challenge. CL is a powerful tool in analytical chemistry that is used for the detection and quantification of reactive oxygen and nitrogen species [24,25], and numerous biological materials such as DNA [26], RNA [27], proteins [28], microorganisms, cells, metals [14,29], etc. [30,31]. Lately, immunoassays have been also used for the detection of SARS-CoV-2 [32].

**Figure 2:**
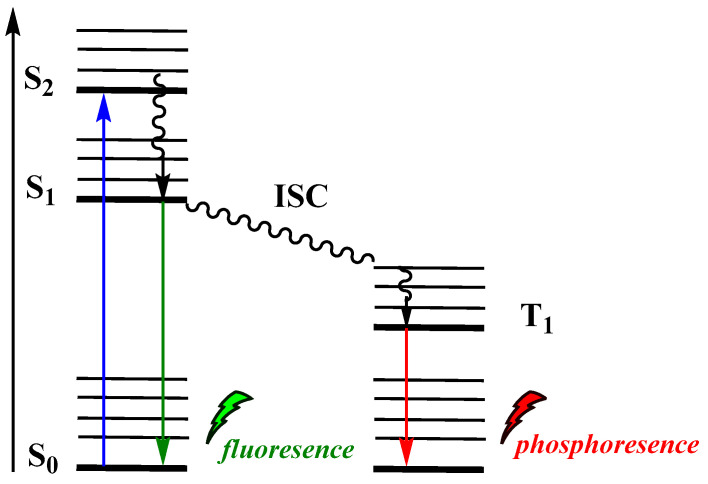
Jablonski diagram showing typical photoexcitation and radiative relaxation.

Moreover, CL has been considered as an alternative photo-uncaging option in drug-delivery technologies for meeting the increasing demand for efficient strategies [15]. However, in biological systems, several issues have to be addressed, such as: (i) the tissue penetration of the incident light used for photoactivation of the emitted photons represents one of major barriers limiting efficiency in imaging or other applications, (ii) light can be scattered or absorbed by other biomolecules inside the cell, and (iii) light intensity is inversely proportional to the square of the distance from the light source, in this way causing weak interaction between an incident photon and a molecule (see Jablonski energy diagram, Figure 2) which leads to poor photochemical interactions. CL has increasingly attracted the interest of the research community, providing solutions to persisting problems of typical photochemical procedures. Recent successful examples from imaging and therapeutics fields highlight the great potential of CL-induced applications. To date, the reported reviews discuss CL mainly focused on chemiluminescent molecules such as luminol, cypridina luciferin and peroxyoxalates or analogues that can be used in sensing, imagine and therapeutic applications [33,34,35,36].

The aim of this review is to provide a summary of the recent developments in this field, based on direct and indirect CL. An overview of the strategies leading to better analytical sensitivity is included along with recent indicative examples. The role of ^1^O_2_ as a chemiexcitation product is also described in relation to the application and further development of more efficient CL-induced therapeutic platforms. Finally, unpublished results of our research regarding the behavior of commonly used photosensitizers as energy acceptors under CL conditions are also included together with discussion on the prospects and the challenges of CL applications in imaging, biomimetic organic and radical chemistry, and therapeutics.

## 2. Mechanistic Aspects of the Types of Chemiluminescence: Direct and Indirect

Among the different types of luminescence, the most typical ones are photoluminescence (PL), chemiluminescence (CL), bioluminescence (BL), and electrochemiluminescence (ECL). A list of recent references for each luminescence type is provided in Table 1.

In PL, incident photons are the driving force for the emission of light by a molecule. Upon absorption of a photon of a particular energy, the molecule becomes photoexcited to an electronically excited state (Figure 2). Among the various paths that can be followed to dissipate the excess energy, radiative relaxation can occur via phosphorescence (T_1_→S_0_) or fluorescence (S_1_→S_0_).

**Figure 3:**
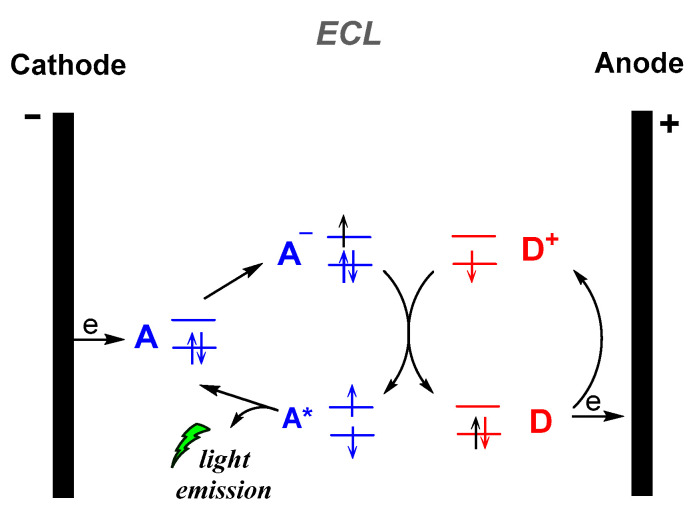
Electrogenerated chemiluminescence (ECL) mechanism.

Electrochemiluminescence (ECL) is the production of light by an excited species that has been generated electrochemically, i.e., an electron transfer reaction at the surface of an electrode (Figure 3). Numerous review articles and monographs are found in the literature to date on the electrogenerated CL, describing the fundamentals, mechanisms and applications. Thus, in this review we will not further discuss this type of CL [42].

In bioluminescence (BL), which is naturally occurring CL, light is emitted by live organisms. Various luminophore molecules, enzymes and cofactors are involved in the more than 30 different bioluminescent systems known to date [40]. As was mentioned before, CL is a process in which chemical reactions can generate an excited intermediate who can emit light directly—direct emission—or transfer its energy to an acceptor fluorophore—indirect emission—(Figure 1). The FRET (Förster Resonance Energy Transfer or Fluorescence Resonance Energy Transfer), which refers to the nonradiative transfer of energy from an excited donor to an acceptor mediated by electronic dipole–dipole coupling, between the oxidized CL reagent (excited state donor) and a fluorophore (acceptor) is referred as chemiluminescence resonance energy transfer (CRET). Apart from bioimaging and sensing applications, recent advances in the PDT field revealed that CL could act as an alternative to conventional light sources. The proximity of the light source to the “target” molecule, as is the case for CL, assists the photochemical transformations to progress faster and photon flux requirements are lower in respect to the procedures based on conventional type of light sources [41].

## 3. Direct Chemiluminescence

**Scheme 1:**
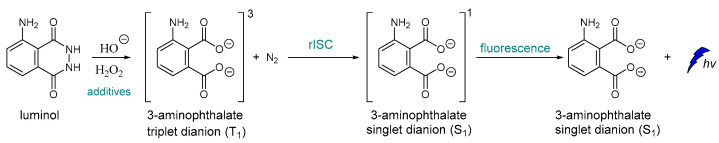
Luminol chemiluminescence mechanism.

To the best of our knowledge, one of the very first examples of direct CL is dated back in 1928 [43], and it is based on the oxidation of luminol by H_2_O_2_ (Scheme 1) [44,45]. After luminol’s oxidation, a strong blue emission with *λ*_max_ = 425 nm is recorded through a reverse intersystem crossing (rISC) [46,47,48] that can last from a second up to few hours, depending on the quantity of the reacting species, the presence of specific additives and the “feeding” process. The light emission of luminol and its derivatives can be assisted by catalysts such as peroxidase and heme, which are usually used as additives. For example, horseradish peroxidase (HRP) is involved in the production of a luminol free radical by two molecules of luminol anion [49].

**Scheme 2:**
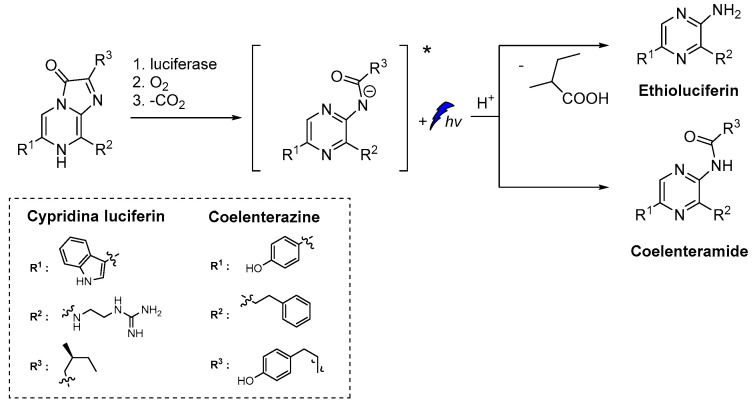
CL of cypridina luciferin, coelenterazine and their derivatives after oxidation [6,7,8,9].

Similarly to luminol, cypridina luciferin emits blue light after oxidation by molecular oxygen catalyzed by cypridina luciferase (Scheme 2) [50,51,52]. Moreover, emission was also recorded after oxidation of 2-methyl-6-phenyl-3,7-dihydroimidazo[1,2-a]pyrazin-3-one, a cypridina luciferin analogue, by O_2_^●–^. At the time of its discovery, this selective oxidation provided a unique opportunity for direct CL detection of O_2_^●–^ and further development of other similar chemiluminescent probes [6].

**Scheme 3:**
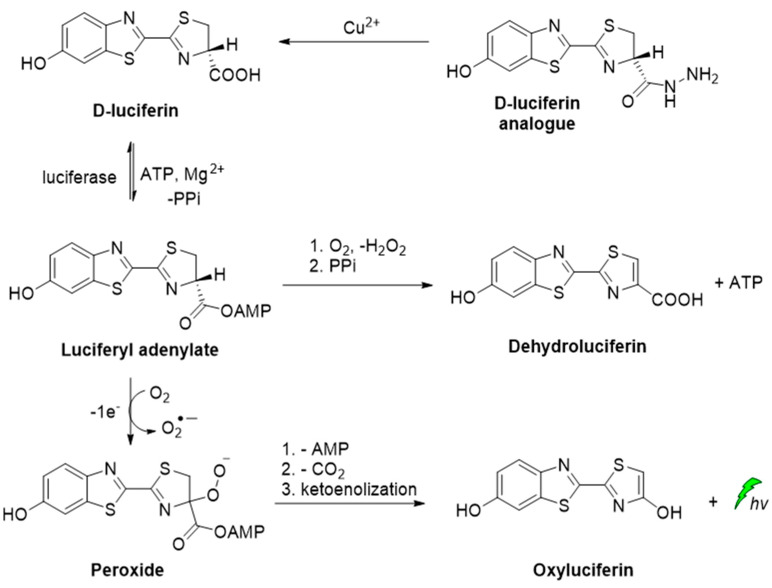
CL of D-luciferin hydrazine analogue catalyzed by Cu^2+^.

A D-luciferin hydrazine analogue has been synthesized for the selective imaging of Cu^2+^. The CL reaction relies on the catalytic transformation of hydrazide analogue to D-luciferin that is further converted to oxyluciferin by luciferase in the presence of ATP and Mg^2+^ (Scheme 3) [53].

Besides the commonly used luminol and cypridina luciferin scaffolds, other compounds such as peroxyoxalates, dioxetane, acridinium and chromone derivatives were also developed and have been used lately as efficient, direct CL reagents in various applications. A summary of recent direct CL applications is provided in Table 2 along with details on the system and the scaffold used.

### 3.1. Direct Chemiluminescence by Luminol and Its Derivatives

**Scheme 4:**
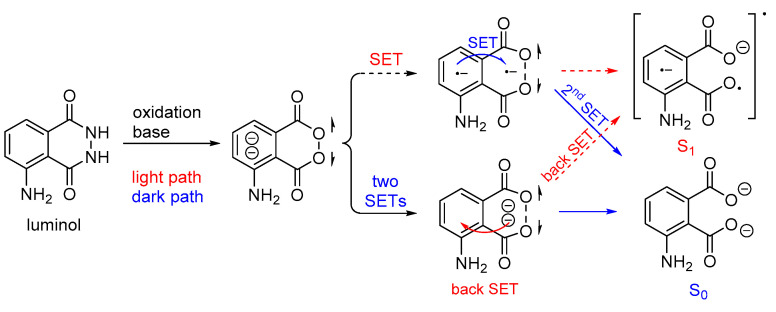
Proposed luminol chemiexcitation mechanism.

In aqueous luminol solutions, H_2_O_2_ usually acts as an oxidant causing CL. However, luminol can also be oxidized catalytically by ozone, ^1^O_2_, and hypochlorites in the presence of various transition metal ions. In contrast, in aprotic solvents (i.e., DMSO) luminol’s CL depends on the pH (basic conditions are required) and the presence of O_2_. Recently, it was proposed that the decomposition of the dianionic cyclic peroxide intermediate (CP^2−^), i.e., 1,2-dioxane-3,6-dione dianion, causes the chemiexcitation of luminol, as shown in Scheme 4. Computational studies have supported a stepwise single-electron transfer (SET) from the amino-phthaloyl to the O−O bond, initiating the decomposition of CP^2−^ [65].

**Figure 4:**
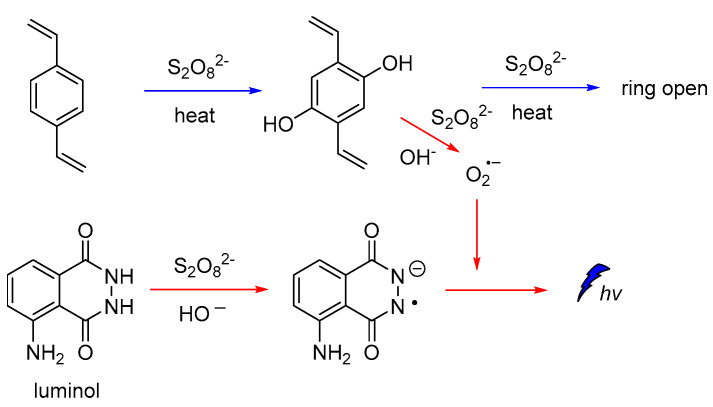
CL mechanism for monitoring hydroxylated intermediates generated on persulfate-based AOPs.

It is also reported that luminol CL could be significantly enhanced in the presence of hydroxylated intermediates generated by persulfate-based advanced oxidation processes (AOPs) of 1,2-divinylbenzene (DVB). Since the emission of light found to be dependent on the oxidation efficiency of the recalcitrant pollutants, luminol CL was proposed (Figure 4) for tracking a wider range of hydroxylated intermediates generated during persulfate-based AOPs under different degradation conditions, and other relevant organic compounds [55].

**Scheme 5:**
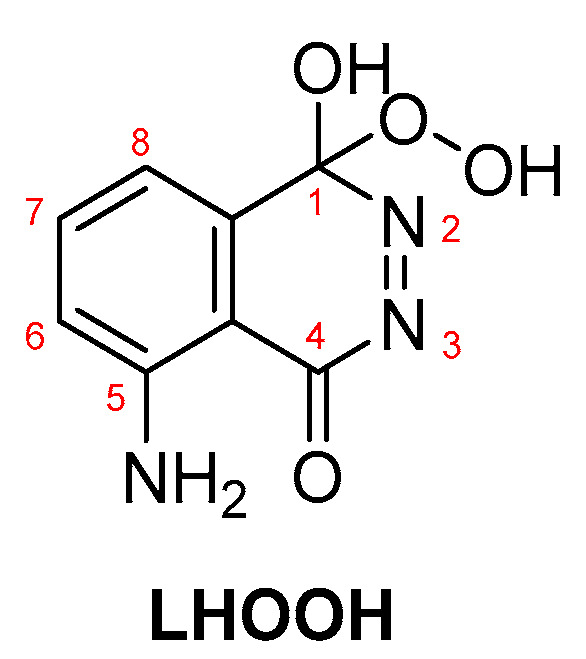
LHOOH intermediate proposed as the key compound for luminol’s CL.

In most cases, where transition metals are involved, metal-peroxide complexes and metal-luminol or metal-luminol-O_2_ intermediates (M-LHOOH) are formed, which are believed to be the key intermediates for the CL of luminol-based systems (Scheme 5) [65].

**Figure 5:**
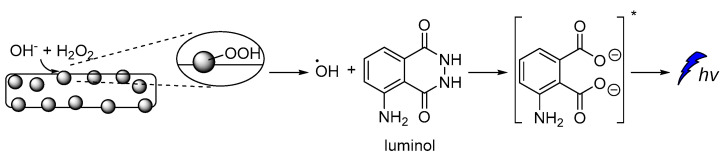
Free radical CL mechanism for the luminol-H_2_O_2_-Co-HAp NPs.

Cobalt-doped hydroxyapatite nanoparticles (CoHA-NPs) have been reported to enhance luminol CL intensity by more than 75 times compared to the conventional luminol-H_2_O_2_ system. During investigations on the impact of CoHA-NPs sizes, composition, and crystallinity on the CL intensity, it was found that the maximum CL intensity is achieved by polycrystalline NPs, owing to their larger surface area. The proposed mechanism involves the ionization of H_2_O_2_ under basic conditions, leading to the formation of hydroperoxide-anions, which subsequently interact with the Co atoms on the NPs surface, forming the metal-hydroperoxide intermediate. The peroxyl radicals, after decomposition of the intermediate, are degraded into HO^•^, which ultimately oxidizes luminol leading to the chemiexcited intermediate (Figure 5) [56].

**Figure 6:**
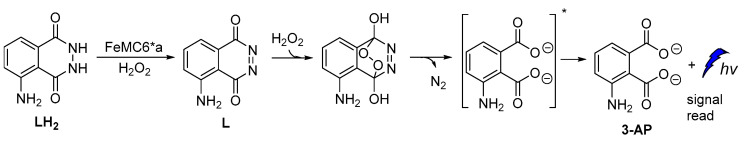
Proposed reaction mechanism of luminol oxidation catalyzed by FeMC6*a.

A new artificial heme-enzyme, so-called FeMC6*a [57], was also found to enhance luminol CL emission signal in comparison to the horseradish peroxidase (HRP)-based sensors (Figure 6), when used in a H_2_O_2_-detecting bioassay [49,66,67,68,69,70,71].

**Figure 7:**
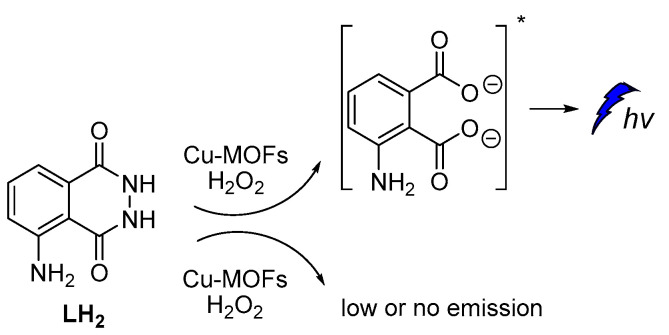
Cu-MOFs catalyzed luminol CL.

The catalytic activity of Cu-metal-organic frameworks (Cu-MOFs) with flower morphology, obtained from Cu-based metal-organic gels, was reported to promote persistent CL in luminol–H_2_O_2_ systems. According to the proposed mechanism, luminol’s CL was attributed to the gradual generation of HO^•^, O_2_^•^‾ and ^1^O_2_ species (Figure 7) and recombination of HO^•^ and O_2_^•^‾ into ^1^O_2_ on the surface of Cu-MOFs, which also resulted in prolonged CL [58].

**Figure 8:**
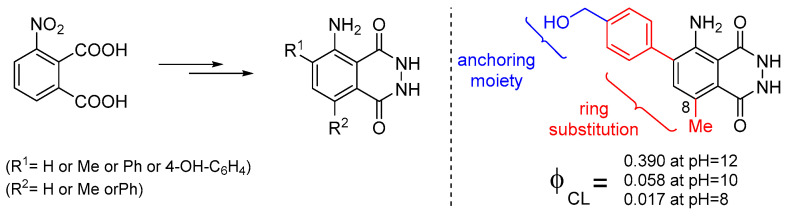
Substitute luminol derivatives with enhanced CL at pH 8, 10 and 12.

Recently, it was also reported that luminol’s CL efficiency can be tuned by manipulating the electronic properties of the aromatic ring via substitution, with the 8-methyl substituent providing the highest *Φ_CL_* enhancement (Figure 8). *In silico* studies revealed that the 8-substitution assists the cleavage of the O-O bond via the negative charge donation to the *in situ* formed peroxide [72]. Previous studies on the factors that could possibly influence luminol’s CL indicated that both steric and electronic effects play a significant role [73].

### 3.2. Direct Chemiluminescence from Probes Other Than Luminol

Apart from luminol derivatives, isoluminol [73], pyridopyridazines and phthalhydrazides [59] that were studied and used as CL probes [74], other molecules have been also designed and used in various CL applications which are not based on luminol’s chemical structure. J. Yang and coworkers reported the synthesis and validation of a new CL molecule, so-called ADLumin-1, as a turn-on probe for amyloid beta (Aβ) species with maximum emission at 590 nm. This probe was then found that can be used in indirect CL via CRET to a curcumin-based NIR fluorescent imaging probe, so-called CRANAD-3, thus more information will be provided in the section on indirect CL (*vide infra*) [60].

**Scheme 6:**
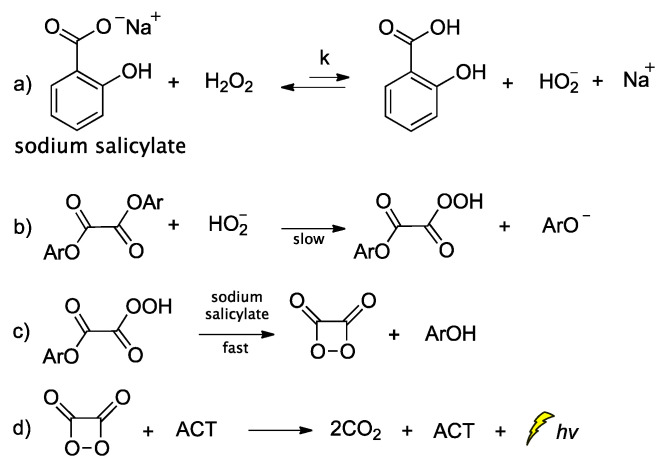
Mechanism of PO-CL between an oxalic ester and H_2_O_2_, occurring via specific base catalysis (SBS) by sodium salicylate, through the formation of (**a**) hydroperoxy anion, (**b**) monoperoxalic ester and (**c**) 1,2-dioxetanedione, which subsequently leads to (**d**) carbon dioxide and light emission.

Peroxyoxalate chemiluminescence (PO-CL) is one of three most common CL reactions [75]. It is a base-catalyzed reaction of an aromatic oxalic ester with H_2_O_2_ in the presence of a fluorophore, also commonly referred to as activator (ACT) [76]. When ACT interacts with the *in situ*-generated high-energy intermediate (HEI), usually a 1,2-dioxetanedione, light emission occurs. The process relies on the reaction between the diphenyloxalate and H_2_O_2_ that generates 1,2-dioxetanedione [77] which spontaneously decomposes to carbon dioxide via a paramagnetic oxalate biradical intermediate [78,79]. Several catalysts have been proposed so far, with imidazole being the most commonly used one [80]. Imidazole acts as a nucleophilic and base catalyst at different reaction steps [81]. Apart from imidazole, only few catalysts appear to work efficiently, and among them are: sodium salicylate, 4-*N*,*N*-dimethylaminopyridine and its derivatives, and a mixture of 1,2,4-triazole and 1,2,2,6,6-pentamethylpiperidine [82,83]. Cabello et al., working on the kinetics of the PO-CL, used three oxalic esters of diverse reactivity (e.g., bis(2,4,6-trichlorophenyl) oxalate (TCPO), bis(4-methylphenyl)oxalate (BMePO), bis[2-(methoxycarbonyl)-phenyl]oxalate (DMO)). The mechanistic studies performed in the presence of sodium salicylate in partially aqueous medium showed that the reaction rate constants were determined by H_2_O_2_ and sodium salicylate amounts (Scheme 6). Further studies showed that by increasing the salicylate concentration there is a decrease in the CL efficiency, probably due to the interaction between salicylate and the in situ-produced HEI. From experiments using different oxalic esters, a variation in the CL emission was reported, with DMO showing the highest quantum yields in the aqueous medium used (1:1 *v*/*v* of DME/H_2_O) [84].

**Scheme 7:**
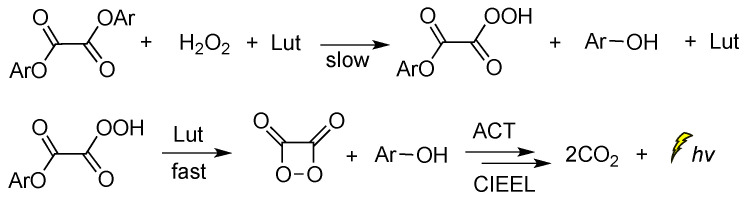
Reaction mechanism of TCPO with H_2_O_2_ in DME under general base catalysis by 2,6-lutidine (Lut) towards CL emission in the presence of a fluorophore.

When 2,6-lutidine was used as a non-nucleophilic base catalyst on the TCPO CL reaction, it was found that the concentrations of 2,6-lutidine and H_2_O_2_ play a crucial role in the rate-determining step. The reaction was found to follow a general base catalysis mechanism (Scheme 7) [85].

**Scheme 8:**
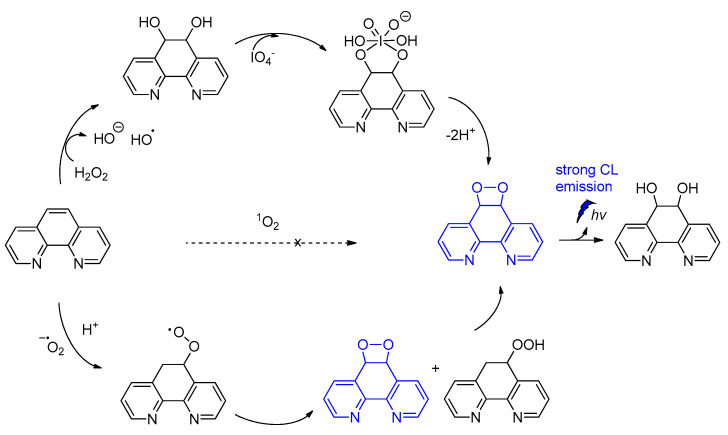
CL of 1,10-phenanthroline dioxetanes.

Among other CL molecules of interest are the 1,10-phenanthroline dioxetanes, generated after treatment of 1,10-phenanthroline with IO_4_^−^ in the presence of H_2_O_2_. It has been reported that 1,10-phenanthroline dioxetanes enhance light emission in respect to the IO_4_^−^-H_2_O_2_ system alone. The proposed radical-based mechanism involves the C^5^=C^6^ bond of 1,10-phenanthroline that is initially attacked by HO^•^, leading to a dioxetane intermediate responsible for the CL emission (Scheme 8) [86]. The CL of the IO_4_^−^/peroxide system and the factors influencing the emission have been recently reviewed elsewhere [87].

**Figure 9:**
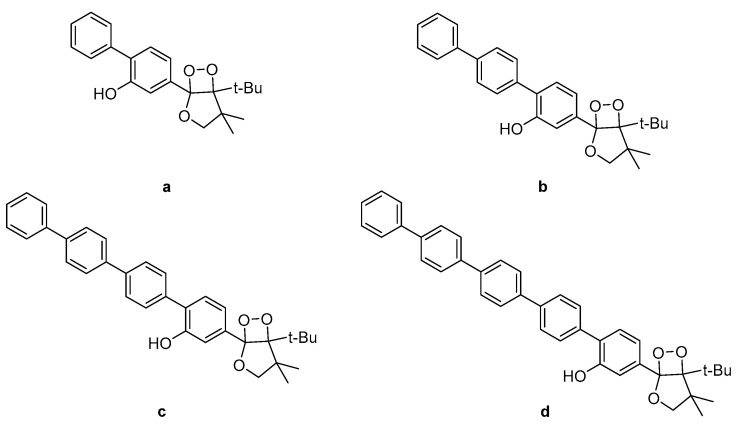
Bicyclic dioxetanes bearing 3-hydroxyphenyl substituted with a p-oligophenylene moiety. (**a**) 2-hydroxybiphenyl-4-yl; (**b**) 2-hydroxy-*p*-terphenyl-4-yl; (**c**) 2-hydroxy-p-quaterphenyl-4-yl; (**d**) 2-hydroxy-*p*-quinquephenyl-4-yl.

Investigation into the CL efficiency of four bicyclic dioxetanes bearing 2-hydroxybiphenyl-4-yl (**a**), 2-hydroxy-*p*-terphenyl-4-yl (**b**), 2-hydroxy-*p*-quaterphenyl-4-yl (**c**), or 2-hydroxy-*p*-quinquephenyl-4-yl (**d**) (Figure 9) moieties, showed that **a-c** emit light from 466 nm to 547 nm during their decomposition while **d** was found to emit light of much weaker intensity from 490 nm up to 607 nm, depending on the solvent (water, acetonitrile or DMSO). The CL efficiencies of the latter dioxetanes in aqueous media were found to be enhanced in the presence of additives such as *β*-MCD (*β*-methylated cyclodextrin) or TBHP (tributylhexadecylphosphonium bromide) [88].

**Figure 10:**
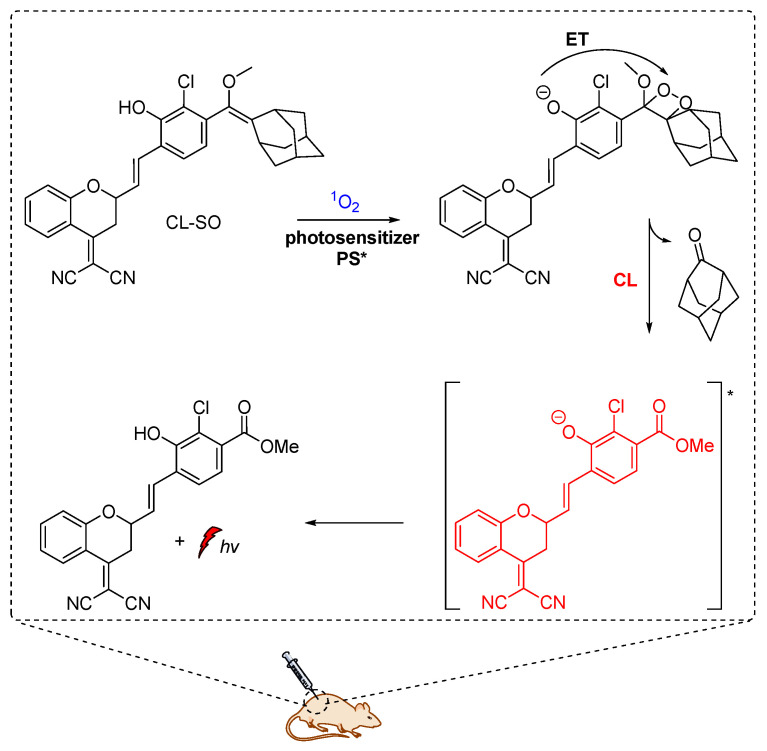
Chemiluminescent probe activated by ^1^O_2_ with maximum emission at 700 nm.

M. Yang et al. constructed a NIR chemiluminescent probe by caging the precursor of Schaap’s dioxetane scaffold and a dicyanomethylchromone acceptor for selective ^1^O_2_ detection. In the presence of ^1^O_2_, the probe is oxidized, affording the phenol-dioxetane moiety which undergoes spontaneous bond cleavage leading to the formation of the chemiexcited product responsible for the CL emission (Figure 10). With this probe, they were able to detect ^1^O_2_ in vitro with a turn-on bathochromic CL signal in the NIR region at 700 nm and image intracellular ^1^O_2_ produced by a photosensitizer under simulated photodynamic therapy conditions [54]. Similarly, hyperconjugated push-pull dicyanomethylchromone systems were used for the *in vivo* detection of H_2_O_2_ and *β*-galactosidase activity [61].

**Scheme 9:**
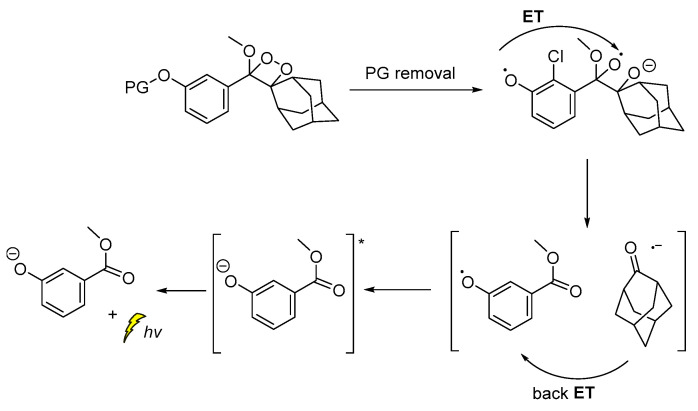
Proposed CIEEL mechanism for triggered CL of Schaap’s dioxetane probes.

The CL emission mechanism proposed for the Schaap’s dioxetane probes [89,90,91], involves Chemically Initiated Electron Exchange Luminescence (CIEEL) [1,92] with a solvent-cage back electron-transfer step, which affords the 2-adamantanone and the chemiexcited phenolate that is responsible for the light emission (Scheme 9) [92,93].

**Scheme 10:**
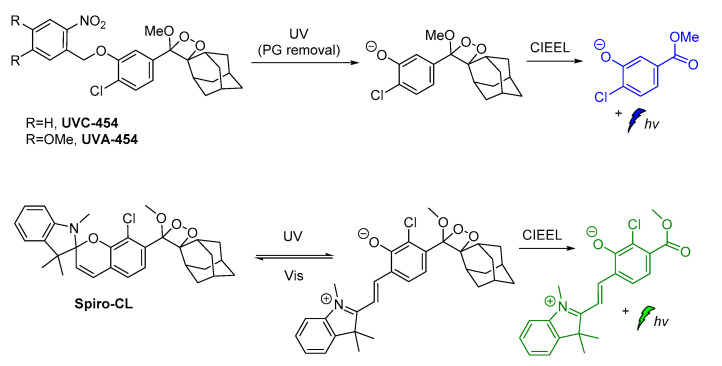
Modified 1,2-dioxetane probes activated by UV light.

Recently, modified ortho-nitrobenzyl and spiropyran 1,2-dioxetane probes have been synthesized and studied towards photoactivation by 254 nm or 365 nm photons. Irradiation of ortho-nitrobenzyl 1,2-dioxetanes with 254 nm light resulted in the formation of the chemiexcited phenolate after deprotection, while the spiropyran 1,2-dioxetane irradiation at *λ =* 254 nm afforded the open merocyanine chromophore intermediate that was finally converted to the corresponding excited phenolate with maximum emission at 500 nm. This open merocyanine intermediate was able to act as a photoswitch, thus it could return to its spiropyran form under white light irradiation subsequently after UV light irradiation (Scheme 10) [94].

**Scheme 11:**
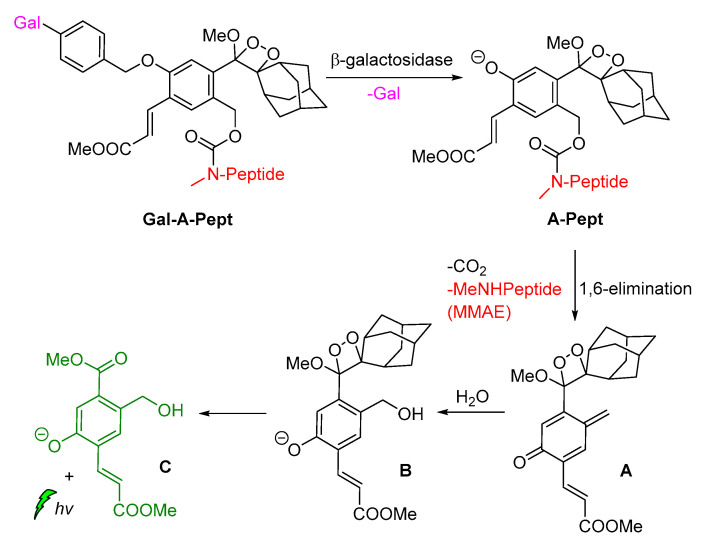
Activation mode of the self-immolative chemiluminescence prodrug **Gal-A-Pept** by *β*-galactosidase.

Shabat and coworkers described the development of functional self-immolative molecular scaffolds based on Schaap’s dioxetane probes, and described their use in signal amplification and real-time monitoring of enzymatic activity or drug release. The general mode of action involves a triggering event for releasing a chemiexcited product, the CL reporter unit, and the payload which can be a reagent molecule, a drug or a prodrug. This strategy was used successfully in monitored release of the cytotoxic agent monomethyl auristatin E (MMAE) from prodrug **Gal-A-Pept** (Scheme 11). The prodrug is initially recognized by a *β*-galactosidase, releasing intermediate **A-Pept** which undergoes 1,6-elimination to afford the MMAE drug and quinone-methide **A**. Then, intermediate **B** is produced by the reaction of **A** with H_2_O, subsequently releasing 2-adamantanone and the chemiexcited phenolate C*. Green emission at 550 nm is observed due to radiative decay of the phenolate to the ground state [62].

**Figure 11:**
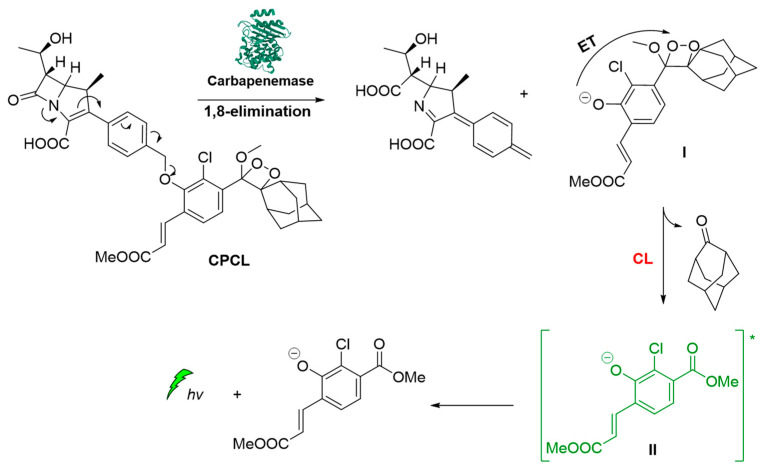
Carbapenemase activity monitoring based on chemiluminescent probe.

Carbapenemase-producing organisms (CPOs) pose a severe threat to antibacterial treatments due to the acquisition of antibiotic resistance. Recently, Das et al. demonstrated the first carbapenemase CL probe (CPCL) for the detection of carbapenemase and CPOs. The unique structural design of CPCL enables the probe to rapidly disassemble via an efficient 1,8-elimination process upon hydrolysis of the *β*-lactam ring by a carbapenemase (Figure 11). This process results in the release of a chemiexcited phenolate that undergoes radiative relaxation, producing photons at 540 nm that enable a rapid and selective detection of carbapenemase activity [63].

The same as above reporter for monitoring carbapenemase activity CL was also used for detection of prostate-specific antigen (PSA) enzymatic activity by using artificial metalloenzymes (ArMs). The probe activation mechanism is based on a catalytic cleavage of a specific peptidyl substrate, followed by a release of a phenoxy-dioxetane intermediate affording a chemiexcited benzoate that emits a green photon. The benefit and simplicity of a CL assay to detect seminal fluid was effectively demonstrated by on-site measurements using a small portable luminometer [64].

**Scheme 12:**
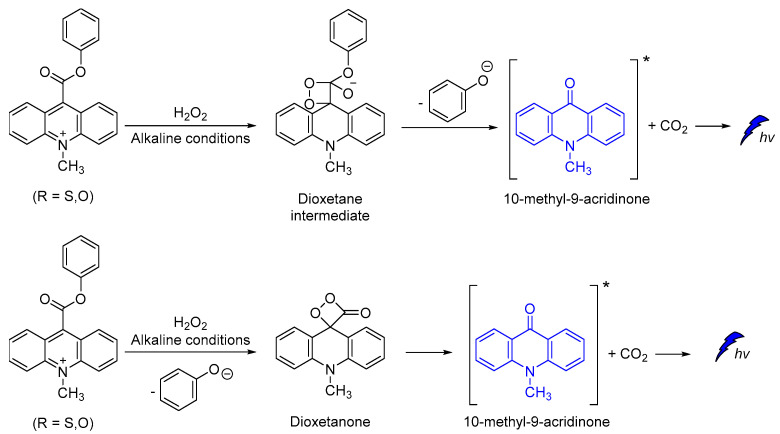
Proposed CL mechanism of acridinium derivatives.

Other efficient CL molecules are based on acridinium ester dyes analogues. The acridan dyes are quaternary acridine derivatives that become luminescent directly after their activation [95,96,97,98,99,100]. The mechanism of CL involving a dioxetane intermediate was first introduced by McCapra (Scheme 12) [101,102]. The simplicity of their synthesis and the high quantum yield (up to ca. 7%) [102], together with their ability to become oxidized under alkaline conditions, making acridinium derivatives highly suitable for sensing and chemiluminometric analysis [95,103]. Research on the design of new acridinium derivatives with even higher activity is an ongoing process [104].

**Scheme 13:**
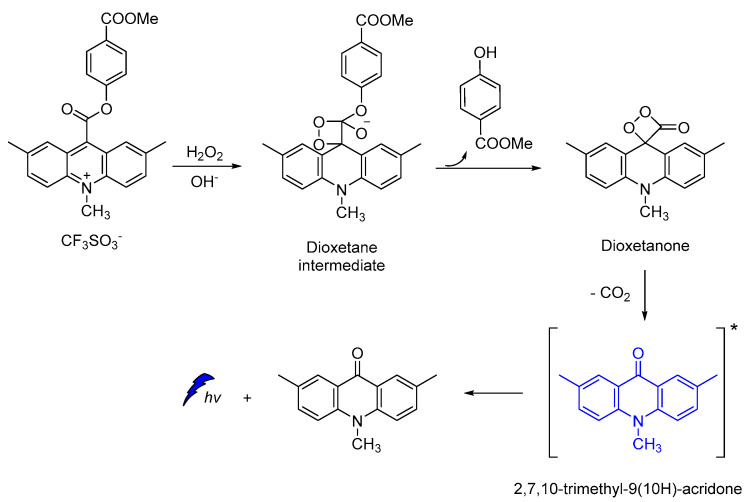
Proposed CL mechanism of 4-(methoxycarbonyl)phenyl-10-methyl-10λ^4^-2,7-dimethyl-acridine-9-carboxylates.

Acridine-9-carboxylate derivatives [e.g., (4-(methoxycarbonyl)phenyl-10-methyl-10λ^4^-2,7-dimethyl-acridine-9-carboxylates)] were used for studying the CL efficiency in the 7–10 pH range. Reactions of acridine-9-carboxylates with H_2_O_2_ result in the formation of a relatively unstable dioxetanone intermediates. The chemiexcited methyl-acridone products formed after the decomposition of the dioxetanone intermediates are responsible for light emission. Among the compounds studied, 4-(methoxycarbonyl)phenyl- derivative gave the highest CL intensities at pH 7−10, indicating that the introduction of electron-donating groups at the 2,7-positions on the acridine moiety plays a crucial role in the whole process (Scheme 13) [105].

## 4. Indirect Chemiluminescence

In the indirect CL process, energy is transferred by the chemiexcited molecule (energy donor, D) to a second molecule (acceptor, E), which is further excited during the process. In the resonance excitation transfer mechanism, D and E are not in contact and may be separated by as much as 10 nm, although 1 nm or smaller transfer distances are more convenient. Except for distance, other factors dictating the energy transfer include the lifetime of the excited state of D and the degree of the overlap between the emission spectrum of D and the absorption spectrum of E. Usually, the higher overlapping the more efficient the energy transfer from D to E. The solvent is another crucial factor for the energy transfer between D and E molecules, however this effect includes a lot of specific characteristics properties of each system, such as the solvent cage factor. The short-range energy transfer by the wavefunction overlap between the donor and the acceptor, meaning overlap between the electronic clouds, is also a crucial factor. Particularly, both the Förster Resonance Energy Transfer or Fluorescence Resonance Energy Transfer (FRET) and Dexter Energy Transfer, with the second occurring when the D–E wavefunctions overlap, contribute to the energy transfer of the system, promoting its scope. FRET, which refers to the nonradiative energy transfer from an excited donor to an acceptor mediated by electronic dipole–dipole coupling, between a CL reagent as the excited donor and a fluorophore acceptor, is referred to as chemiluminescence resonance energy transfer (CRET) [106].

**Scheme 14:**
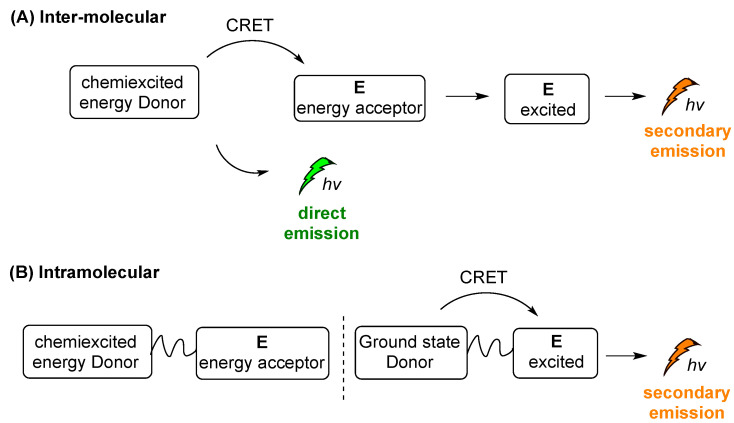
Intermolecular (**A**) and intramolecular (**B**) indirect CL emission modes.

Two different modes of indirect CL emission can be distinguished, intermolecular and intramolecular CL, as shown in a simplified manner in Scheme 14A,B, respectively. After the triggering event, chemiexcited D is formed and then its energy is transferred to E, e.g., a photosensitizer (PS). When E is not directly linked with the chemiexcited species (Scheme 14A), the intermolecular distance between D and E (photosensitizer) plays crucial role in the energy transfer, with a direct emission (“escaping” photons) taking place together with the secondary emission. Sometimes this mode of action is crucial for distinguishing the two different events and their efficacy rate: one of the initial CL emission (direct emission) and the second is processed from the excited acceptor properties (Scheme 14A, secondary emission). On the other hand, in the case of an intramolecular system, the chemiexcited donor (D) is linked with the energy acceptor molecule (E) and a secondary emission pathway is favored (Scheme 14B).

The absorbance band of conventional photosensitizers such as Rose Bengal and TetraPhenylPorphyrin (TPP) (Type II photosensitizers) [107,108,109,110], as well as W_10_O_32_^4−^ (Type I’ photocatalyst) [111,112,113], fit well into the requirements for efficient CRET and high CL emissions [114]. Quantum Dots (QDs) have been also used successfully in various CRET studies. QDs are luminescent semiconductor nanocrystals with very distinct, size-dependent physicochemical behavior [106]. It should be noted that the excited photosensitizer or QDs can trigger various reaction cascades. For example, in the presence of molecular oxygen they can result in the production of ROS (e.g., ^1^O_2_, HO^●^, O_2_^●−^). This function is used in CL-induced photodynamic therapies and is described later in the text in more details (*vide infra*) [115].

A summary of recent direct CL scaffolds, systems and their applications are provided in Table 3 and Table 4, based on intra- and inter-molecular modes of reaction.

### 4.1. Intermolecular Chemiluminescence Emission Mode

**Figure 12:**
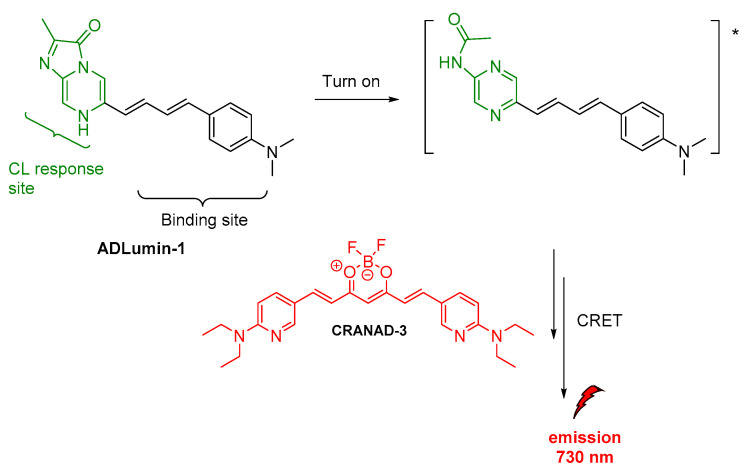
Indirect CL from **ADLumin−1**/**CRANAD-3** system.

J. Yang and coworkers, as mentioned above (*vide supra*), reported on the ADLumin-1 probe for Aβ species (Figure 12). To overcome problems related to short emission of ADLumin-1 they used the CRET strategy for transferring the energy to **CRANAD-3**, a curcumin-based, NIR fluorescent imaging probe emitting in the near-infrared region, at 730 nm (*vide supra*) [60].

**Figure 13:**
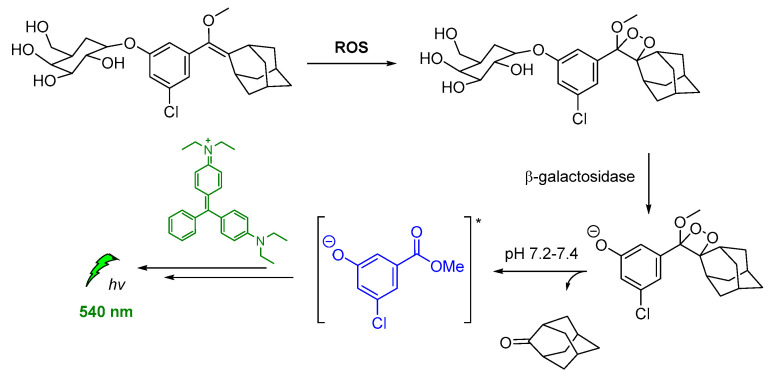
Indirect CL via energy transfer from activated by *β*-galactosidase 1,2-dioxetane to the Emerald^TM^ enhancer emitting at 540 nm.

Spiroadamantane 1,2-dioxetanes have been used in CL assay for *in vivo* visualization of *β*-galactosidase enzymatic activity in transgenic mice. For achieving this, an accelerant (diethanolamine in buffer containing EmeraldTM enhancer, T2081) was administered along with the *β*-D-galactopyranoside trigger (3-chloro-5-(5′-chloro-4-methoxyspiro[1,2-dioxetane-3,2′-tricyclo[3.3.1.13,7]decan]-4-yl)phenyl *β*-D-galacto pyranoside Galacton Plus). Thus, the energy was transferred by the produced chemiexcited intermediate to the Emerald^TM^ accelerant, causing emission at 540 nm (Figure 13) [120].

**Figure 14:**
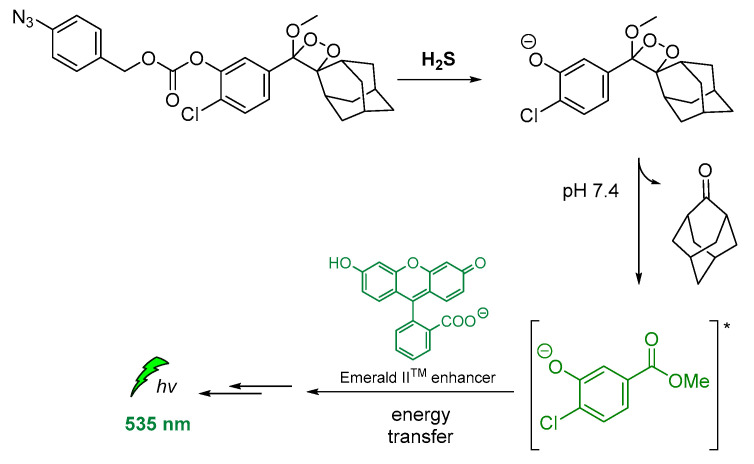
Indirect CL via energy transfer from H_2_S-activated 1,2-dioxetane to the Emerald II^TM^ enhancer emitting at 535 nm.

Other modified 1,2-dioxetane probes were also synthesized and used for *in vivo* imaging of hydrogen sulfide (H_2_S). The reduction of the azide on the *para*-azidobenzyl carbonate moiety triggered the self-immolative cleavage, affording the chemiexcited phenolate. Then, the energy transfer via CRET to the Emerald II^TM^ CL enhancer [121] allowed detection at 545 nm (Figure 14) [122].

**Figure 15:**
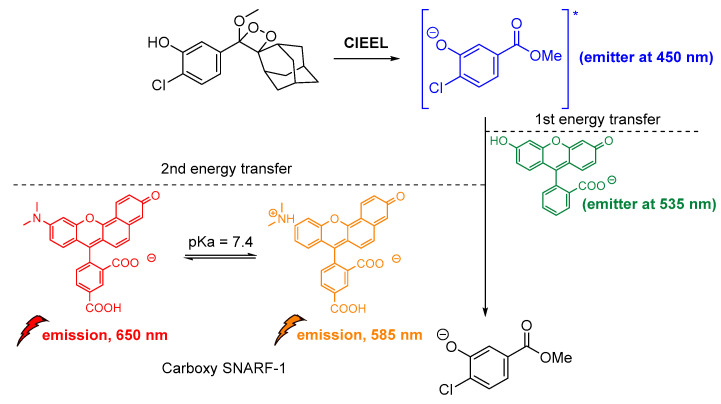
Indirect CL via energy transfer from 1,2-dioxetane to the Emerald II^TM^ enhancer and subsequently to the carboxy-SNARF-1 dye (pKa = 7.4), emitting at 585 nm and 650 nm depending on the pH.

Another important study by Lipper et al., described that CL energy transfer from Schaap’s dioxetane probe can be efficiently used for ratiometric pH imaging. Their approach was based on energy transfer after the emission of phenolate during radiative relaxation to the pH-sensitive dye carboxy-SNARF-1 in CL-enhancer solutions, providing thee relative intensities at 535 nm, 585 nm and 650 nm due to relaxation of the protonated and deprotonated dye, respectively (Figure 15) [116].

Recently, a supramolecular strategy was reported, achieving a highly efficient CL-initiated cascade FRET by following a three-criteria rule: (i) D emission and the E absorption spectral overlapping; (ii) 10 nm or less as the D–E distance; and (iii) orientation of the D and E dipoles (see also Scheme 14). For this purpose, a nanoassemble *β*-cyclodextrin (*β*-CD) was used in an aqueous luminol/H_2_O_2_ solution in the presence of various fluorophores, which enable an accelerated multicolor CL with flexible emission wavelength in the range of 410–610 nm. Hydroxypropyl methylcellulose was utilized to slow down the diffusion rate, while the reaction was buffered by addition of solid Ca(OH)_2_. Moreover, it was found that Co^2+^-chitosan increased the CL emission of the system when merged with the *β*-CD nanoassembly system [126].

**Figure 16:**
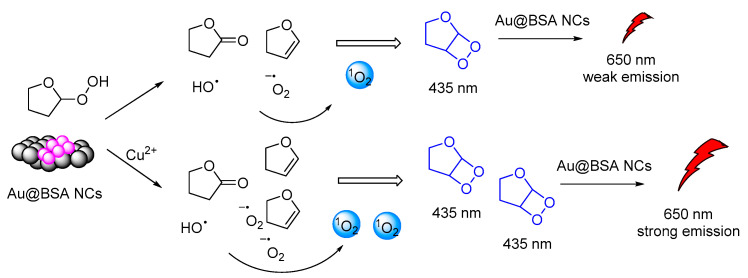
CL mechanism of the Au@BSA NCs-THF-CPO-Cu^2+^ system.

Another interesting CL system of cyclic peroxides was catalytically produced by tetrahydrofuran hydrogen peroxide in the presence of BSA-stabilized Gold Nanoclusters (Au@BSA NCs). Gold nanoclusters were found to accelerate the decomposition of the THF-hydroperoxides and promoted CRET between the chemiexcited dioxetane derivatives and the Au@BSA NCs (Figure 16). The CL emission (*λ*_max_ = 650 nm) was found to be further enhanced by the presence of copper ions [127].

From the investigation into the CL behavior of an amphiphilic conjugate (defined as CLP) NPs, it was revealed that the NPs consisting of chlorine6 (Ce6), conjugated with luminol and polyethylene glycol undergo CRET with energy transfer to Ce6 upon triggering with H_2_O_2_. The CLP NPs were tested in cancer cells with high expression of H_2_O_2_ and monitored at 660–740 nm. In addition, excited Ce6 was capable of producing ^1^O_2_ in situ PDT against H_2_O_2_-high tumors, inhibiting lung metastasis. *In vitro* and *in vivo* experiments showed that CLP NPs could be a promising system in nanotheranostics, providing imaging and therapy, simultaneously, particularly in tumors with high levels of H_2_O_2_ [128].

Black Phosphorene (BP) QDs were initially studied in CL experiments upon reaction with H_2_O_2_ and HClO by Y. Lv and coworkers [129]. The same group two years later reported on the extension of the study with NH_2_-functionalized BP QDs (N-BPQDs) and showed that surface modification dictates the energy gap and electron mobility, thus favoring CL emission at 500 nm after triggering by persulfates [130].

Luminol’s analogue, L012, that shows 100 times higher than luminol CL intensity after oxidation by H_2_O_2_ at pH 7, was also used as a CRET donor. *In vitro* and *in vivo* studies with polyenthylene(glycol)-coated CdSeTe core QDs, used as energy acceptors, demonstrated that they can image H_2_O_2_ in live cells, pointing out the high potential of the system in the diagnosis of H_2_O_2_-associated diseases. The chemiexcited QDs were able to emit light at 780 nm, thus allowing monitoring [117].

**Figure 17:**
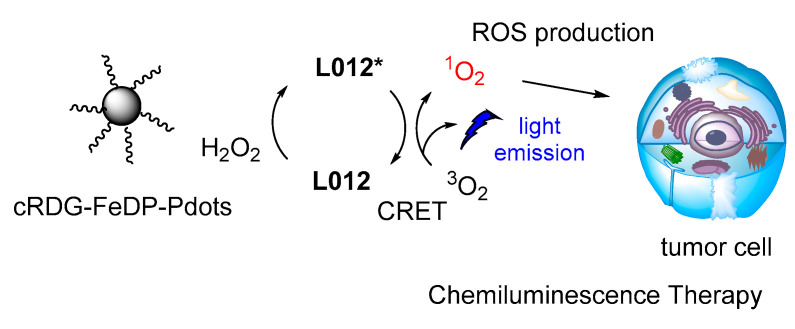
CL dynamic therapy based on L012/H_2_O_2_ catalyzed by FeDP-Pdots.

The same luminol analogue L012 and H_2_O_2_ were recently studied in the presence of Fe(III) deuteroporphyrin IX chloride-polymer dots (FeDP-Pdots) as CL catalysts towards cancer therapy, providing high concentrations of ROS due to high CRET efficiency (Figure 17) [118].

**Figure 18:**
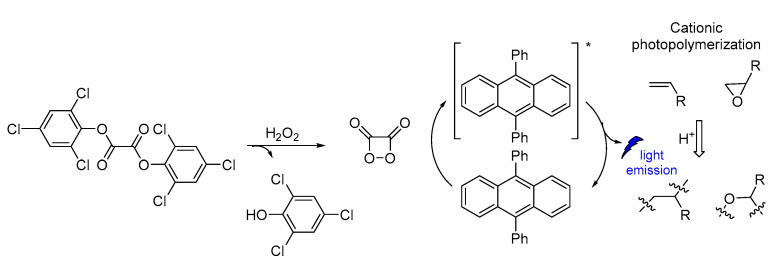
CL induced cationic polymerization by using sulfonium salt as a photoinitiator.

CL has been also used in polymerization reactions. Linear polymer chains were synthesized along with cross-linked functional polymeric networks as a result of CL photoinitiated cationic polymerization based on a sulfonium salt (Figure 18). The polymerization reaction initiated CL emission at 430 nm. The photoinitiator decomposition after excitation produced protonic acids that were capable of initiating cationic polymerization of oxirane (epoxides) and vinyl monomers. The presence of iodonium salt in the initiating system was found to increase the polymerization efficiency [119].

### 4.2. Intramolecular Chemiluminescence Emission Mode

**Figure 19:**
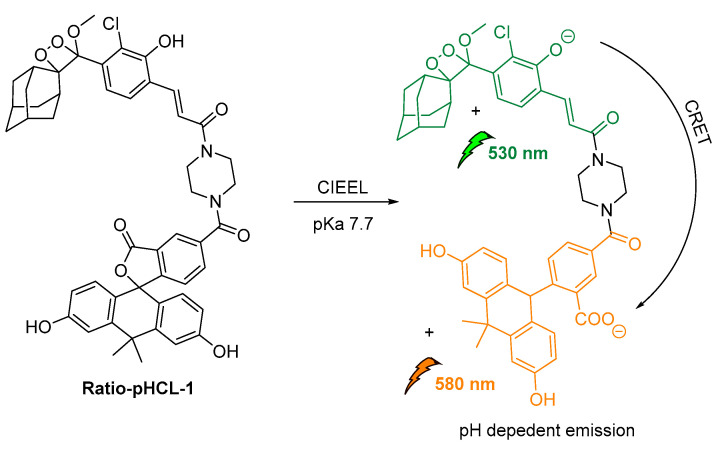
Design and mechanism of ratio-pHCL-1 (CIEEL).

An intramolecular CRET approach based on chemically initiated electron exchange luminescence (CIEEL) has been recently adapted, while developing the first single molecule ratiometric CL probe for imaging pH in live animals. The proposed direct–indirect emission hybrid methodology, with emissions at 530 nm and 580 nm, relies on an acrylamide 1,2-dioxetane scaffold linked via a piperazine linker to a pH-sensitive carbofluorescein. This methodology withholds many characteristics that make it suitable for applications in quantifications of other important analytes produced in vivo (Figure 19) [123].

**Scheme 15:**
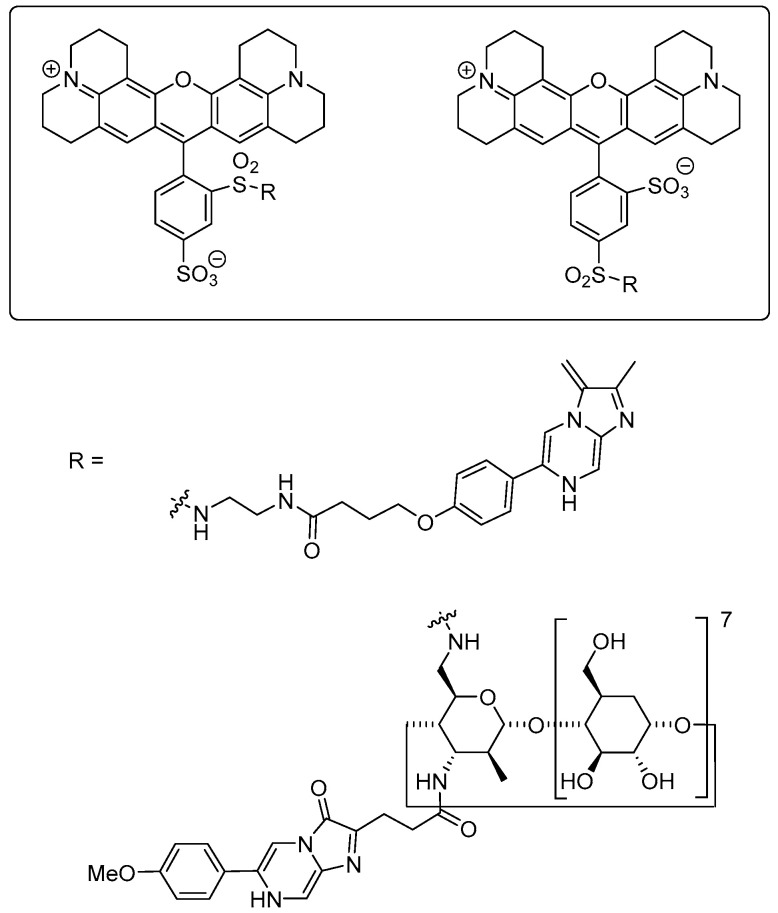
Chemical structures of chemiluminescent probes emitting light at 610 nm.

Teranishi proposed a cypridina luciferin analogue for detecting O_2_^•−^ via intramolecular CRET to sulforhodamine 101. The energy transfer from the oxidized imidazopyrazinone to the acceptor dye results in light emission at 610 nm (Scheme 15). A similar approach was studied earlier with fluorescein analogue FCLA, emitting at 532 nm [124].

**Figure 20:**
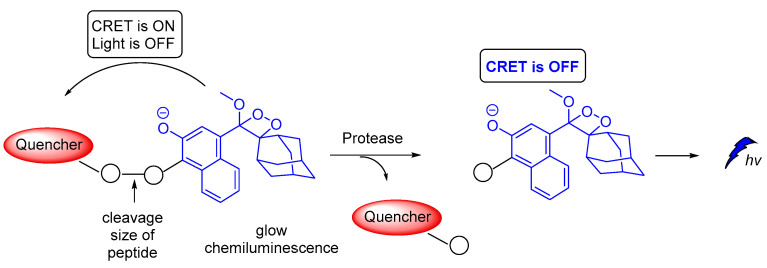
Chemiluminescence activation mechanism of a Glow-CRET system.

A novel on–off CRET system based on phenoxy-dioxetane 7-hydroxycoumarin scaffold for light emission, upon slow chemiexcitation in aqueous solution, was recently reported. The energy donor is covalently linked to a quencher via a peptide linker that is recognizable by metalloproteinases. In the absence of enzymatic activity CRET is on, thus the energy is transferred to the quencher. When the peptide linker is cleaved by enzymes CRET is off, thus light emission is observed (Figure 20). The system was tested in cancer cells, showing good detection levels in matrix metalloproteinase activity [125].

## 5. The Dual Role of Singlet Oxygen in Chemiluminescence

**Scheme 16:**
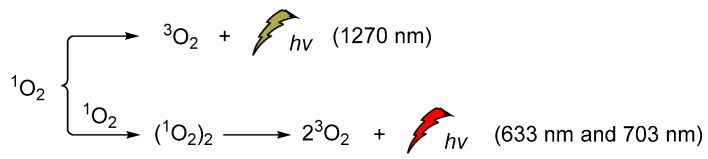
^1^O_2_ chemiluminescence with emissions at 633 nm, 703 nm and 1270 nm.

Singlet oxygen was discovered and confirmed spectroscopically in the early 1960s when the H_2_O_2_/hypochlorite system was at the epicenter of the CL studies [131,132,133,134,135,136,137,138]. Its emission at 1270 nm was attributed to the monomeric singlet oxygen and the emissions at 633 nm and 703 nm, to its dimeric aggregate form (Scheme 16). It is noteworthy that the energy difference between the ground state of O_2_ (^3^Σ_g_) and its singlet excited state (^1^Δ_g_, singlet oxygen, ^1^O_2_) is 22.5 kcal/mol. Moreover, its lifetime depends significantly on the solvent (e.g., ~59 ms in carbon tetrachloride, 9.8 ± 0.6 μs in methanol-h_4_, 77 ± 4 μs acetonitrile and 3.7 ± 0.4 μs in water) [139,140].

**Figure 21:**
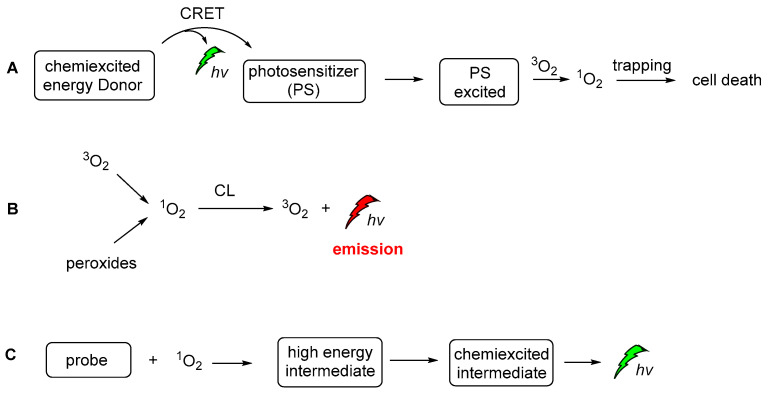
The CL-induced ^1^O_2_ production (**A**), the CL emission produced by the radiative decay of singlet oxygen (**B**) and CL emission after the activation of a probe molecule by ^1^O_2_ (**C**).

During recent decades, an increasing number of studies have focused on exploring the role of ^1^O_2_ in chemistry, biology, medicine and environmental and material sciences. Lately, the increasing interest of the scientific community in novel therapies has led to further exploration of ^1^O_2_ against persisting diseases (i.e., cancer). Thus, a significant amount of the new studies are now focused on the generation modes and the photophysical properties [141], dictating its interaction with other biomolecules in live cells. On the other hand, ^1^O_2_ is a very important oxidizing agent in organic chemistry transformations. There are two main approaches for generating ^1^O_2_. The first, and more famous one, involves light and a photosensitizer, while the less famous one is hydroperoxide-based and it is often called the “in dark” process, since ^1^O_2_ is produced in the absence of light. To avoid confusion between the CL-induced ^1^O_2_production and the CL of the ^1^O_2_ itself, we should distinguish here the two completely different CL. The energy transfer and radiative processes of the CL energy acceptors or chemiexcited intermediates are illustrated in Figure 21.

The majority of the recent photochemical or CL studies on ^1^O_2_ applications employ well-known and established methodologies for generating an efficient amount of the oxidizing agent based on photosensitizers such as TTP, Rose Bengal, Cu^2+^ [142], Ru^2+^ and Ir^3+^ complexes, phthalocyanines [143,144] and other analogues. Kellett and his coworkers have presented a class of di-copper (II) complexes based on the synthetic chemical nuclease [Cu(Phen)_2_]^+^ (where Phen = 1,10-phenathroline), that were selective against solid epithelial cancer cells from line panel NCI-60. Among other ROS, their agents are found to catalyze intracellular ^1^O_2_ contributing to oxidative DNA damage [145]. Recently, our team has also explored the role of Cu^+^ complexes in the photochemical production of ^1^O_2_. We found and presented for the first time that [Cu(Xantphos)(neoc)]BF_4_ can be used as an alternative and very efficient photosensitizer in ^1^O_2_-mediated C–H allylic oxygenations [146].

**Scheme 17:**
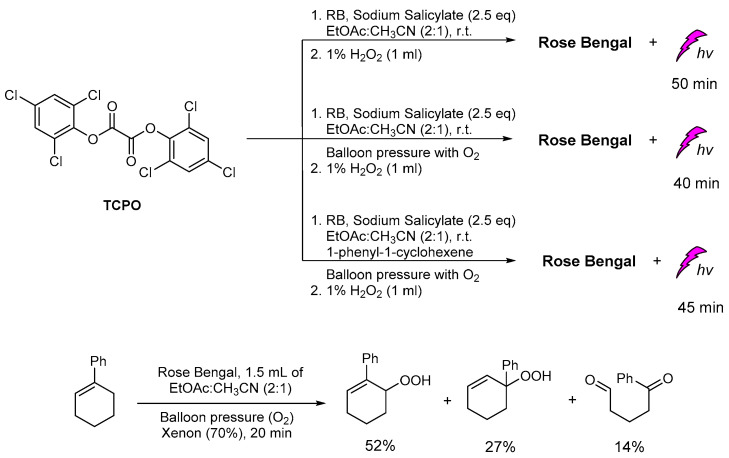
The CL-induced singlet oxygen production for the oxidation of 1-phenylcyclohexene in “green” solvent ethyl acetate.

Recently, in our group, the plausible production of ^1^O_2_ was studied under different CL reaction conditions and in the presence of various sensitizers. In this study, except for the common solvents used in CL procedures, diethylphthalates, the greener ethyl acetate was found to give unexpectedly good results. In addition, several photosensitizers, i.e., Rose Bengal (RB), Methylene Blue, 9,10-diphenylanthracene (9,10-DPA), Eosin Y and Rhodamine B, were tested for light emission through the CL process, with RB providing the best results based on the light emission duration. In the presence of TCPO, H_2_O_2_, RB, sodium salicylate and a mixture of ethyl acetate/acetonitrile in a ratio of 2/1, light emission was observed for 40–50 min (Scheme 17). Under these CL conditions and in the presence of 1-phenylcycloalkene, no allylic oxidation product was observed. For comparison, the same solution was bubbled with molecular oxygen and irradiated using a Xenon lamp (300 W, λ > 320 nm) [147,148,149], however, no oxidation products were observed. In the absence of TCPO and H_2_O_2_, the corresponding alkene was oxidized leading to the corresponding allylic hydroperoxides via a ^1^O_2_ oxidation pathway, within 20 minutes (Scheme 17). These results indicate that the observed CL phenomenon didn’t induce ^1^O_2_ production, or the energy transfer and radiative processes of the CL energy acceptors or chemiexcited intermediates are not enough for the oxidation of cycloalkene. Further experiments using different photosensitizers and initial alkenes are in progress to determine this new intermolecular oxidation pathway [150].

## 6. Future Prospects

Various technologies have emerged during the past few years aiming to fine-tune the molecular scaffolds and the experimental conditions under which CL can be used for the development of more advanced molecular sensors and new materials, in drug delivery, theranostics and polymerization reactions. To that end, several scaffolds such as dioxetanes and luminol-based systems, etc., have already shown great potential and are expected to play a significant role in the years to come. The growing demand for highly efficient PDT platforms that could produce significant amounts of reactive species under CL conditions may also reveal another important research direction that is still unexplored, regarding the replacement of light sources by CL in standard photochemical reactions.

A summary of the major obstacles that cause delay in the expansion of the CL applications include, but are not limited to, (i) the photophysical properties of the CL probe such as the fluorescence quantum yield, emission wavelength, photon flux, CL kinetic profile; (ii) the solubility and stability of the CL probe in the reaction media; (iii) the distance between the energy donor and energy acceptor, plus their emission and absorption spectral overlapping, in the case of direct CL; (iv) the selectivity of the triggering agent and oxidation mechanism of the CL substrates; (v) the efficiency of the emission mechanism (i.e., CIEEL); (vi) the toxicity, when considering bioapplications.

Recently there has been a tremendous increase in the number of new photochemical reactions reported in the literature. Nonetheless, reports of industrial applications utilizing this new knowledge are still scarce and mainly limited to flow-chemistry processes. Maybe nature can inspire the research community to find a solution to this problem through CL?

## Figures and Tables

**Table 1:** Types of luminescence based on different activation mode.

Type of Luminescence	Activation Mode	References
Photoluminescence (PL)	Photoexcitation	[37,38,39]
Chemiluminescence (CL) and Bioluminescence (BL)	Chemiexcitation	[40,41]
Electrochemiluminescence (ECL)	Electrochemical Excitation	[42]

**Table 2:** Summary of recent scaffolds, systems and applications based on direct chemiluminescence [54].

Scaffold	System	Applications	References
Luminol	Without System, Cobalt-Doped Hydroxyapatite Nanoparticles, Cu-Metal-Organic Frameworks	Monitoring of Advanced Oxidation Processes, Sensing, H_2_O_2_ and O_2_^●–^ Detection	[55,56,57,58,59]
ADLumin-1		Amyloid Beta (Aβ) Species Probe	[60]
Dioxetane	Precursor of Dioxetane Containing the Dicyanomethylchromone Moiety, Functional Self-immolative Molecular Scaffolds	^1^O_2_ Detection in vitro and in vivo, Monitoring of *β*-Galactosidase Activity—Drug Uncaging, Carbapenemase Activity, Enzymatic Activity of Prostate Specific Antigen (PSA)	[54,61,62,63,64]

**Table 3:** Summary of recent scaffolds, systems and applications based on intermolecular indirect chemiluminescence.

Scaffold	System	Applications	References
ADLumin-1	CRANAD-3	Aβ Species Probe	[60]
Luminol	Chlorine6 Containing QDs Polymeric NPs, Fe(III) Deuteroporphyrin IX Chloride NPs, CdSeTe Core QDs	H_2_O_2_ Imaging and Therapy	[116,117,118]
Peroxyoxalate	9,10-Diphenylanthracene	Cationic Polymerization	[119]
Dioxetane	Emerald^TM^ and Emerald II^TM^ Enhancers, Carboxy-SNARF-1 Dye	Imaging of *β*-Galactosidase and Nitroreductase Activities, H_2_S, Ratiometric pH	[116,120,121,122]

**Table 4:** Summary of imaging, sensing and therapy approaches based on intramolecular indirect chemiluminescence.

Scaffold	System	Applications	References
Dioxetane	Molecular pH-Sensitive Carbofluorescein Probe Precursor of Dioxetane	Ratiometric pH Imaging in Live Animals	[123]
Cypridina Luciferin	Sulforhodamine 101	O_2_^•−^ Detection	[124]
Phenoxy-Dioxetane 7-Hydroxycoumarin	FRET Quencher	Monitoring of Matrix Metalloproteinase Activity	[125]

## Data Availability

Not applicable.

## References

[1] Vacher M., Fdez Galván I., Ding B.-W., Schramm S., Berraud-Pache R., Naumov P., Ferré N., Liu Y.-J., Navizet I., Roca-Sanjuán D. (2018). Chemi- and Bioluminescence of Cyclic Peroxides. Chem. Rev..

[2] McCapra F. (1990). Chemiluminescence and Bioluminescence. J. Photochem. Photobiol. A Chem..

[3] Viviani V.R. (2002). The Origin, Diversity, and Structure Function Relationships of Insect Luciferases. CMLS Cell. Mol. Life Sci..

[4] White E.H., McCapra F., Field G.F., McElroy W.D. (1961). The structure and synthesis of firefly luciferin. J. Am. Chem. Soc..

[5] Yagur-Kroll S., Bilic B., Belkin S. (2010). Strategies for Enhancing Bioluminescent Bacterial Sensor Performance by Promoter Region Manipulation: Enhancing Bacterial Sensor Performance. Microb. Biotechnol..

[6] Goto T., Takagi T. (1980). Chemiluminescence of a *Cypridina* Luciferin Analogue, 2-Methyl-6-Phenyl-3,7-Dihydroimidazo[1,2- *a* ]Pyrazin-3-One, in the Presence of the Xanthine–Xanthine Oxidase System. BCSJ.

[7] Toya Y., Kayano T., Sato K., Goto T. (1992). Synthesis and Chemiluminescence Properties of 6-(4-Methoxyphenyl)-2-Methylimidazo[1,2-*a*]Pyrazin-3(7*H*)-One and 2-Methyl-6-(2-Naphthyl)Imidazo[1,2-*a*]Pyrazin-3(7*H*)-One. BCSJ.

[8] Teranishi K. (2007). Development of Imidazopyrazinone Red-Chemiluminescent Probes for Detecting Superoxide Anions via a Chemiluminescence Resonance Energy Transfer Method. Luminescence.

[9] Sekiya M., Umezawa K., Sato A., Citterio D., Suzuki K. (2009). A Novel Luciferin-Based Bright Chemiluminescent Probe for the Detection of Reactive Oxygen Species. Chem. Commun..

[10] Marques S.M., Esteves da Silva J.C.G. (2009). Firefly Bioluminescence: A Mechanistic Approach of Luciferase Catalyzed Reactions. IUBMB Life.

[11] Kim J.E., Kalimuthu S., Ahn B.-C. (2015). In Vivo Cell Tracking with Bioluminescence Imaging. Nucl. Med. Mol. Imaging.

[12] Brown J.E., De Weer P., Salzberg B.M. (2020). Optical Measurement of Changes in Intracellular Calcium. Biophys. J..

[13] Adam W., Kazakov D.V., Kazakov V.P. (2005). Singlet-Oxygen Chemiluminescence in Peroxide Reactions. Chem. Rev..

[14] Gnaim S., Shabat D. (2019). Activity-Based Optical Sensing Enabled by Self-Immolative Scaffolds: Monitoring of Release Events by Fluorescence or Chemiluminescence Output. Acc. Chem. Res..

[15] Haris U., Kagalwala H.N., Kim Y.L., Lippert A.R. (2021). Seeking Illumination: The Path to Chemiluminescent 1,2-Dioxetanes for Quantitative Measurements and In Vivo Imaging. Acc. Chem. Res..

[16] Chatgilialoglu C., Ferreri C., Krokidis M.G., Masi A., Terzidis M.A. (2021). On the Relevance of Hydroxyl Radical to Purine DNA Damage. Free. Radic. Res..

[17] Chatgilialoglu C., Krokidis M.G., Masi A., Barata-Vallejo S., Ferreri C., Terzidis M.A., Szreder T., Bobrowski K. (2019). New Insights into the Reaction Paths of Hydroxyl Radicals with Purine Moieties in DNA and Double-Stranded Oligodeoxynucleotides. Molecules.

[18] Chatgilialoglu C., Ferreri C., Terzidis M.A. (2011). Purine 5′,8-Cyclonucleoside Lesions: Chemistry and Biology. Chem. Soc. Rev..

[19] Terzidis M.A., Chatgilialoglu C. (2015). An Ameliorative Protocol for the Quantification of Purine 5′,8-Cyclo-2′-Deoxynucleosides in Oxidized DNA. Front. Chem..

[20] McCaughan J.S. (1999). Photodynamic Therapy: A Review. Drugs Aging.

[21] Dolmans D.E., Fukumura D., Jain R.K. (2003). Photodynamic Therapy for Cancer. Nat. Rev. Cancer.

[22] Zhao X., Liu J., Fan J., Chao H., Peng X. (2021). Recent Progress in Photosensitizers for Overcoming the Challenges of Photodynamic Therapy: From Molecular Design to Application. Chem. Soc. Rev..

[23] Gunaydin G., Gedik M.E., Ayan S. (2021). Photodynamic Therapy for the Treatment and Diagnosis of Cancer–A Review of the Current Clinical Status. Front. Chem..

[24] Yamaguchi S., Kishikawa N., Ohyama K., Ohba Y., Kohno M., Masuda T., Takadate A., Nakashima K., Kuroda N. (2010). Evaluation of Chemiluminescence Reagents for Selective Detection of Reactive Oxygen Species. Anal. Chim. Acta.

[25] Worsfold P., Townshend A., Poole C.F. (2005). Encyclopedia of Analytical Science.

[26] Qi Y., Xiu F., Weng Q. (2018). A Novel and Convenient Chemiluminescence Sensing of DNA: Nanometer Interface Effect and DNA Action Mechanism. Sens. Actuators B Chem..

[27] Ling K., Jiang H., Huang X., Li Y., Lin J., Li F.-R. (2018). Direct Chemiluminescence Detection of Circulating MicroRNAs in Serum Samples Using a Single-Strand Specific Nuclease-Distinguishing Nucleic Acid Hybrid System. Chem. Commun..

[28] Li W., Zhang Q., Zhou H., Chen J., Li Y., Zhang C., Yu C. (2015). Chemiluminescence Detection of a Protein through the Aptamer-Controlled Catalysis of a Porphyrin Probe. Anal. Chem..

[29] Carter K.P., Young A.M., Palmer A.E. (2014). Fluorescent Sensors for Measuring Metal Ions in Living Systems. Chem. Rev..

[30] Roda A., Pasini P., Musiani M., Girotti S., Baraldini M., Carrea G., Suozzi A. (1996). Chemiluminescent Low-Light Imaging of Biospecific Reactions on Macro- and Microsamples Using a Videocamera-Based Luminograph. Anal. Chem..

[31] Roda A., Pasini P., Musiani M., Baraldini M. (2000). Chemiluminescence Imaging Systems for the Analysis of Macrosamples: Microtiter Format, Blot Membrane, and Whole Organs. Methods in Enzymology.

[32] Padoan A., Cosma C., Sciacovelli L., Faggian D., Plebani M. (2020). Analytical Performances of a Chemiluminescence Immunoassay for SARS-CoV-2 IgM/IgG and Antibody Kinetics. Clin. Chem. Lab. Med. (CCLM).

[33] Yan Y., Wang X., Hai X., Song W., Ding C., Cao J., Bi S. (2020). Chemiluminescence Resonance Energy Transfer: From Mechanisms to Analytical Applications. TrAC Trends Anal. Chem..

[34] Yan Y., Shi P., Song W., Bi S. (2019). Chemiluminescence and Bioluminescence Imaging for Biosensing and Therapy: In Vitro and In Vivo Perspectives. Theranostics.

[35] Delafresnaye L., Bloesser F.R., Kockler K.B., Schmitt C.W., Irshadeen I.M., Barner-Kowollik C. (2020). All Eyes on Visible-Light Peroxyoxalate Chemiluminescence Read-Out Systems. Chem. Eur. J..

[36] Hananya N., Shabat D. (2019). Recent Advances and Challenges in Luminescent Imaging: Bright Outlook for Chemiluminescence of Dioxetanes in Water. ACS Cent. Sci..

[37] Albini A. (2015). Photochemistry: Past, Present and Future.

[38] Crespi S., Protti S. (2021). Photochemistry: Volume 49.

[39] Powe A.M., Fletcher K.A., St. Luce N.N., Lowry M., Neal S., McCarroll M.E., Oldham P.B., McGown L.B., Warner I.M. (2004). Molecular Fluorescence, Phosphorescence, and Chemiluminescence Spectrometry. Anal. Chem..

[40] Shimomura O. (2006). Bioluminescence: Chemical Principles and Methods.

[41] Magalhães C.M., Esteves da Silva J.C.G., Pinto da Silva L. (2016). Chemiluminescence and Bioluminescence as an Excitation Source in the Photodynamic Therapy of Cancer: A Critical Review. ChemPhysChem.

[42] Miao W. (2008). Electrogenerated Chemiluminescence and Its Biorelated Applications. Chem. Rev..

[43] Albrecht H.O. (1928). Über Die Chemiluminescenz Des Aminophthalsäurehydrazids. Z. Phys. Chem..

[44] Vaughan W.R. (1948). The Chemistry of the Phthalazines. Chem. Rev..

[45] White E.H., Zafiriou O., Kagi H.H., Hill J.H. (1964). Chemilunimescence of Luminol: The Chemcial Reaction. J. Am. Chem. Soc..

[46] Yu Y., Mallick S., Wang M., Börjesson K. (2021). Barrier-Free Reverse-Intersystem Crossing in Organic Molecules by Strong Light-Matter Coupling. Nat Commun..

[47] Wada Y., Nakagawa H., Matsumoto S., Wakisaka Y., Kaji H. (2020). Organic Light Emitters Exhibiting Very Fast Reverse Intersystem Crossing. Nat. Photonics.

[48] Cui L.-S., Gillett A.J., Zhang S.-F., Ye H., Liu Y., Chen X.-K., Lin Z.-S., Evans E.W., Myers W.K., Ronson T.K. (2020). Fast Spin-Flip Enables Efficient and Stable Organic Electroluminescence from Charge-Transfer States. Nat. Photonics.

[49] Nakamura M., Nakamura S. (1998). One- and Two-Electron Oxidations of Luminol by Peroxidase Systems. Free. Radic. Biol. Med..

[50] Morin J.G. (2011). Based on a Review of the Data, Use of the Term ‘Cypridinid’ Solves the Cypridina/Vargula Dilemma for Naming the Constituents of the Luminescent System of Ostracods in the Family Cypridinidae. Luminescence.

[51] Johnson F.H., Shimomura O. (1978). Introduction to the Cypridina System. Methods Enzymol..

[52] Khakhar A., Starker C.G., Chamness J.C., Lee N., Stokke S., Wang C., Swanson R., Rizvi F., Imaizumi T., Voytas D.F. (2020). Building customizable auto-luminescent luciferase-based reporters in plants. Elife.

[53] Zheng Z., Wang L., Tang W., Chen P., Zhu H., Yuan Y., Li G., Zhang H., Liang G. (2016). Hydrazide D-Luciferin for in Vitro Selective Detection and Intratumoral Imaging of Cu^2+^. Biosens. Bioelectron..

[54] Yang M., Huang J., Fan J., Du J., Pu K., Peng X. (2020). Chemiluminescence for Bioimaging and Therapeutics: Recent Advances and Challenges. Chem. Soc. Rev..

[55] Li Z., Chen X., Teng X., Lu C. (2019). Chemiluminescence as a New Indicator for Monitoring Hydroxylated Intermediates in Persulfate-Based Advanced Oxidation Processes. J. Phys. Chem. C.

[56] Vakh C., Kuzmin A., Sadetskaya A., Bogdanova P., Voznesenskiy M., Osmolovskaya O., Bulatov A. (2020). Cobalt-Doped Hydroxyapatite Nanoparticles as a New Eco-Friendly Catalyst of Luminol–H2O2 Based Chemiluminescence Reaction: Study of Key Factors, Improvement the Activity and Analytical Application. Spectrochim. Acta Part A Mol. Biomol. Spectrosc..

[57] Zambrano G., Nastri F., Pavone V., Lombardi A., Chino M. (2020). Use of an Artificial Miniaturized Enzyme in Hydrogen Peroxide Detection by Chemiluminescence. Sensors.

[58] Yang C.P., He L., Huang C.Z., Li Y.F., Zhen S.J. (2021). Continuous Singlet Oxygen Generation for Persistent Chemiluminescence in Cu-MOFs-Based Catalytic System. Talanta.

[59] Nishinaka Y., Aramaki Y., Yoshida H., Masuya H., Sugawara T., Ichimori Y. (1993). A New Sensitive Chemiluminescence Probe, L-012, for Measuring the Production of Superoxide Anion by Cells. Biochem. Biophys. Res. Commun..

[60] Yang J., Yin W., Van R., Yin K., Wang P., Zheng C., Zhu B., Ran K., Zhang C., Kumar M. (2020). Turn-on Chemiluminescence Probes and Dual-Amplification of Signal for Detection of Amyloid Beta Species in Vivo. Nat Commun..

[61] Green O., Gnaim S., Blau R., Eldar-Boock A., Satchi-Fainaro R., Shabat D. (2017). Near-Infrared Dioxetane Luminophores with Direct Chemiluminescence Emission Mode. J. Am. Chem. Soc..

[62] Gnaim S., Scomparin A., Das S., Blau R., Satchi-Fainaro R., Shabat D. (2018). Direct Real-Time Monitoring of Prodrug Activation by Chemiluminescence. Angew. Chem. Int. Ed..

[63] Das S., Ihssen J., Wick L., Spitz U., Shabat D. (2020). Chemiluminescent Carbapenem-Based Molecular Probe for Detection of Carbapenemase Activity in Live Bacteria. Chem. Eur. J..

[64] Gutkin S., Green O., Raviv G., Shabat D., Portnoy O. (2020). Powerful Chemiluminescence Probe for Rapid Detection of Prostate Specific Antigen Proteolytic Activity: Forensic Identification of Human Semen. Bioconjug. Chem..

[65] Yue L., Liu Y.-T. (2020). Mechanistic Insight into PH-Dependent Luminol Chemiluminescence in Aqueous Solution. J. Phys. Chem. B.

[66] Kamidate T., Katayama A., Ichihashi H., Watanabe H. (1994). Characterization of Peroxidases in Luminol Chemiluminescence Coupled with Copper-Catalysed Oxidation of Cysteamine. J. Biolumin. Chemilumin..

[67] Gorsuch J.D., Hercules D.M. (1972). Studies on the chemiluminescence of luminol in dimethylsulfoxide and dimethylsulfoxide-water mixtures. Photochem. Photobiol..

[68] Lee J., Seliger H.H. (1972). Quantum yields of the luminol chemiluminescence reaction in aqueous and aprotic solvents. Photochem. Photobiol..

[69] Cormier M.J., Prichard P.M. (1968). An Investigation of the Mechanism of the Luminescent Peroxidation of Luminol by Stopped Flow Techniques. J. Biol. Chem..

[70] Li L., Arnold M.A., Dordick J.S. (1993). Mathematical Model for the Luminol Chemiluminescence Reaction Catalyzed by Peroxidase: Peroxidase-catalyzed luminol chemiluminescence. Biotechnol. Bioeng..

[71] Cercek B., Cercek B., Roby K., Cercek L. (1994). Effect of Oxygen Abstraction on the Peroxidase-Luminol-Perborate System: Relevance to the HRP Enhanced Chemiluminescence Mechanism. J. Biolumin. Chemilumin..

[72] Mikroulis T., Cuquerella M.C., Giussani A., Pantelia A., Rodríguez-Muñiz G.M., Rotas G., Roca-Sanjuán D., Miranda M.A., Vougioukalakis G.C. (2021). Building a Functionalizable, Potent Chemiluminescent Agent: A Rational Design Study on 6,8-Substituted Luminol Derivatives. J. Org. Chem..

[73] Brundrett R.B., White E.H. (1974). Synthesis and Chemiluminescence of Derivatives of Luminol and Isoluminol. J. Am. Chem. Soc..

[74] Karatani H. (1987). Microenvironmental Effects of Water-Soluble Polymers on the Chemiluminescence of Luminol and Its Analogs. BCSJ.

[75] Baader W.J., Stevani C.V., Bastos E.L., Rappoport Z. (2009). Chemiluminescence of Organic Peroxides The Authors Dedicate This Chapter to the Late Prof. Dr. Giuseppe Cilento (Universidade de São Paulo), Whose Untimely Demise in 1994 Saddened Everyone Who Knew Him as an Excellent Scientist and Outstanding Human Being. This Chapter Is Also Dedicated to Prof. Waldemar Adam (Universität Würzburg, Now ‘Retired’ in Puerto Rico), Who Was Directly or Indirectly Responsible for the Research Interests of the Authors. PATAI’S Chemistry of Functional Groups.

[76] Tonkin S.A., Bos R., Dyson G.A., Lim K.F., Russell R.A., Watson S.P., Hindson C.M., Barnett N.W. (2008). Studies on the Mechanism of the Peroxyoxalate Chemiluminescence Reaction. Anal. Chim. Acta.

[77] Farahani P., Baader W.J. (2017). Unimolecular Decomposition Mechanism of 1,2-Dioxetanedione: Concerted or Biradical? That Is the Question!. J. Phys. Chem. A.

[78] Bos R., Tonkin S.A., Hanson G.R., Hindson C.M., Lim K.F., Barnett N.W. (2009). In Search of a Chemiluminescence 1,4-Dioxy Biradical. J. Am. Chem. Soc..

[79] Orosz G. (1989). The Role of Diaryl Oxalates in Peroxioxalate Chemiluminescence. Tetrahedron.

[80] Stevani C.V., Lima D.F., Toscano V.G., Baader W.J. (1996). Kinetic Studies on the Peroxyoxalate Chemiluminescent Reaction: Imidazole as a Nucleophilic Catalyst. J. Chem. Soc. Perkin Trans. 2.

[81] Silva S.M., Casallanovo F., Oyamaguchi K.H., Ciscato L.F., Stevani C.V., Baader W.J. (2002). Kinetic Studies on the Peroxyoxalate Chemiluminescence Reaction: Determination of the Cyclization Rate Constant. Luminescence.

[82] Souza G.A., Lang A.P., Baader W.J. (2017). Mechanistic Studies on the Peroxyoxalate Chemiluminescence Using Sodium Salicylate as Base Catalyst. Photochem. Photobiol..

[83] Jonsson T., Irgum K. (2000). New Nucleophilic Catalysts for Bright and Fast Peroxyoxalate Chemiluminescence. Anal. Chem..

[84] Cabello M.C., Souza G.A., Bello L.V., Baader W.J. (2020). Mechanistic Studies on the Salicylate-Catalyzed Peroxyoxalate Chemiluminescence in Aqueous Medium. Photochem. Photobiol..

[85] Augusto F.A., Bartoloni F.H., Cabello M.C., dos Santos A.P.F., Baader W.J. (2019). Kinetic Studies on 2,6-Lutidine Catalyzed Peroxyoxalate Chemiluminescence in Organic and Aqueous Medium: Evidence for General Base Catalysis. J. Photochem. Photobiol. A Chem..

[86] Shah S.N.A., Shah A.H., Dou X., Khan M., Lin L., Lin J.-M. (2019). Radical-Triggered Chemiluminescence of Phenanthroline Derivatives: An Insight into Radical–Aromatic Interaction. ACS Omega.

[87] Shah S.N.A., Khan M., Rehman Z.U. (2020). A Prolegomena of Periodate and Peroxide Chemiluminescence. TrAC Trends Anal. Chem..

[88] Watanabe N., Takatsuka H., Ijuin H.K., Matsumoto M. (2020). Highly Effective and Rapid Emission of Light from Bicyclic Dioxetanes Bearing a 3-Hydroxyphenyl Substituted with a 4-p-Oligophenylene Moiety in an Aqueous System: Two Different Ways for the Enhancement of Chemiluminescence Efficiency. Tetrahedron.

[89] Schaap A.P., Chen T.-S., Handley R.S., DeSilva R., Giri B.P. (1987). Chemical and Enzymatic Triggering of 1,2-Dioxetanes. 2: Fluoride-Induced Chemiluminescence from Tert-Butyldimethylsilyloxy-Substituted Dioxetanes. Tetrahedron Lett..

[90] Schaap A.P., Handley R.S., Giri B.P. (1987). Chemical and Enzymatic Triggering of 1,2-Dioxetanes. 1: Aryl Esterase-Catalyzed Chemiluminescence from a Naphthyl Acetate-Substituted Dioxetane. Tetrahedron Lett..

[91] Schaap A.P., Sandison M.D., Handley R.S. (1987). Chemical and Enzymatic Triggering of 1,2-Dioxetanes. 3: Alkaline Phosphatase-Catalyzed Chemiluminescence from an Aryl Phosphate-Substituted Dioxetane. Tetrahedron Lett..

[92] Augusto F.A., de Souza G.A., de Souza Júnior S.P., Khalid M., Baader W.J. (2013). Efficiency of Electron Transfer Initiated Chemiluminescence. Photochem. Photobiol..

[93] Adam W., Bronstein I., Trofimov A.V., Vasil’ev R.F. (1999). Solvent-Cage Effect (Viscosity Dependence) as a Diagnostic Probe for the Mechanism of the Intramolecular Chemically Initiated Electron-Exchange Luminescence (CIEEL) Triggered from a Spiroadamantyl-Substituted Dioxetane. J. Am. Chem. Soc..

[94] Ryan L.S., Nakatsuka A., Lippert A.R. (2021). PhotoactivaTable 1,2-Dioxetane Chemiluminophores. Results Chem..

[95] Zadykowicz B., Czechowska J., Ożóg A., Renkevich A., Krzymiński K. (2016). Effective Chemiluminogenic Systems Based on Acridinium Esters Bearing Substituents of Various Electronic and Steric Properties. Org. Biomol. Chem..

[96] Czechowska J., Kawecka A., Romanowska A., Marczak M., Wityk P., Krzymiński K., Zadykowicz B. (2017). Chemiluminogenic Acridinium Salts: A Comparison Study. Detection of Intermediate Entities Appearing upon Light Generation. J. Lumin..

[97] Nakazono M., Nanbu S., Akita T., Hamase K. (2019). Synthesis, Chemiluminescence, and Application of 2,4-Disubstituted Phenyl 10-Methyl-10λ4-Acridine-9-Carboxylates. Dyes Pigments.

[98] Ren L., Cui H. (2014). Chemiluminescence Accompanied by the Reaction of Acridinium Ester and Manganese (II): Reaction of Acridinium Ester and Manganese(II). Luminescence.

[99] Natrajan A., Wen D. (2014). Effect of Branching in Remote Substituents on Light Emission and Stability of Chemiluminescent Acridinium Esters. RSC Adv..

[100] Krzymiński K., Ożóg A., Malecha P., Roshal A.D., Wróblewska A., Zadykowicz B., Błażejowski J. (2011). Chemiluminogenic Features of 10-Methyl-9-(Phenoxycarbonyl)Acridinium Trifluoromethanesulfonates Alkyl Substituted at the Benzene Ring in Aqueous Media. J. Org. Chem..

[101] McCapra F. (1966). The Chemiluminescence of Organic Compounds. Q. Rev. Chem. Soc..

[102] Weeks I., Beheshti I., McCapra F., Campbell A.K., Woodhead J.S. (1983). Acridinium Esters as High-Specific-Activity Labels in Immunoassay. Clin. Chem..

[103] Natrajan A., Sharpe D. (2013). Synthesis and Properties of Differently Charged Chemiluminescent Acridinium Ester Labels. Org. Biomol. Chem..

[104] Pieńkos M., Zadykowicz B. (2020). Computational Insights on the Mechanism of the Chemiluminescence Reaction of New Group of Chemiluminogens—10-Methyl-9-Thiophenoxycarbonylacridinium Cations. IJMS.

[105] Nakazono M., Nanbu S., Akita T., Hamase K. (2020). Chemiluminescence of Methoxycarbonylphenyl 10-Methyl-10λ4 -2,7-Disubstituted Acridine-9-Carboxylate Derivatives. J. Photochem. Photobiol. A Chem..

[106] Huang X., Li L., Qian H., Dong C., Ren J. (2006). A Resonance Energy Transfer between Chemiluminescent Donors and Luminescent Quantum-Dots as Acceptors (CRET). Angew. Chem. Int. Ed..

[107] Tanielian C., Mechin R., Seghrouchni R., Schweitzer C. (2000). Mechanistic and Kinetic Aspects of Photosensitization in the Presence of Oxygen^†§^. Photochem. Photobiol..

[108] Greer A. (2006). Christopher Foote’s Discovery of the Role of Singlet Oxygen [^1^O_2_ (^1^Δ_g_)] in Photosensitized Oxidation Reactions. Acc. Chem. Res..

[109] Stratakis M., Orfanopoulos M. (1997). Regioselectivity in the Ene Reaction of Singlet Oxygen with Alkenes Bearing an Electron Withdrawing Group at β- Position. Tetrahedron Lett..

[110] You Y. (2018). Chemical Tools for the Generation and Detection of Singlet Oxygen. Org. Biomol. Chem..

[111] Tanielian C. (1998). Decatungstate Photocatalysis. Coord. Chem. Rev..

[112] Tzirakis M.D., Lykakis I.N., Orfanopoulos M. (2009). Decatungstate as an Efficient Photocatalyst in Organic Chemistry. Chem. Soc. Rev..

[113] Ravelli D., Protti S., Fagnoni M. (2016). Decatungstate Anion for Photocatalyzed “Window Ledge” Reactions. Acc. Chem. Res..

[114] Meyer S., Tietze D., Rau S., Schäfer B., Kreisel G. (2007). Photosensitized Oxidation of Citronellol in Microreactors. J. Photochem. Photobiol. A Chem..

[115] Samia A.C.S., Chen X., Burda C. (2003). Semiconductor Quantum Dots for Photodynamic Therapy. J. Am. Chem. Soc..

[116] An W., Mason R.P., Lippert A.R. (2018). Energy Transfer Chemiluminescence for Ratiometric PH Imaging. Org. Biomol. Chem..

[117] Lee E.S., Deepagan V.G., You D.G., Jeon J., Yi G.-R., Lee J.Y., Lee D.S., Suh Y.D., Park J.H. (2016). Nanoparticles Based on Quantum Dots and a Luminol Derivative: Implications for in Vivo Imaging of Hydrogen Peroxide by Chemiluminescence Resonance Energy Transfer. Chem. Commun..

[118] Teng Y., Li M., Huang X., Ren J. (2020). Singlet Oxygen Generation in Ferriporphyrin-Polymer Dots Catalyzed Chemiluminescence System for Cancer Therapy. ACS Appl. Bio Mater..

[119] Wang C., Meng X., Li Z., Li M., Jin M., Liu R., Yagci Y. (2020). Chemiluminescence Induced Cationic Photopolymerization Using Sulfonium Salt. ACS Macro Lett..

[120] Liu L., Mason R.P. (2010). Imaging β-Galactosidase Activity in Human Tumor Xenografts and Transgenic Mice Using a Chemiluminescent Substrate. PLoS ONE.

[121] Cao J., Campbell J., Liu L., Mason R.P., Lippert A.R. (2016). In Vivo Chemiluminescent Imaging Agents for Nitroreductase and Tissue Oxygenation. Anal. Chem..

[122] Cao J., Lopez R., Thacker J.M., Moon J.Y., Jiang C., Morris S.N.S., Bauer J.H., Tao P., Mason R.P., Lippert A.R. (2015). Chemiluminescent Probes for Imaging H_2_S in Living Animals. Chem. Sci..

[123] Ryan L.S., Gerberich J., Haris U., Nguyen D., Mason R.P., Lippert A.R. (2020). Ratiometric PH Imaging Using a 1,2-Dioxetane Chemiluminescence Resonance Energy Transfer Sensor in Live Animals. ACS Sens..

[124] Suzuki N., Suetsuna K., Mashiko S., Yoda B., Nomoto T., Toya Y., Inaba H., Goto T. (1991). Reaction Rates for the Chemiluminescence of *Cypridina* Luciferin Analogues with Superoxide: A Quenching Experiment with Superoxide Dismutase. Agric. Biol. Chem..

[125] Hananya N., Press O., Das A., Scomparin A., Satchi-Fainaro R., Sagi I., Shabat D. (2019). Persistent Chemiluminescent Glow of Phenoxy-dioxetane Luminophore Enables Unique CRET-Based Detection of Proteases. Chem. Eur. J..

[126] Song Q., Yan X., Cui H., Ma M. (2020). Efficient Cascade Resonance Energy Transfer in Dynamic Nanoassembly for Intensive and Long-Lasting Multicolor Chemiluminescence. ACS Nano.

[127] Zhang K., Sun M., Song H., Su Y., Lv Y. (2020). Synergistic Chemiluminescence Nanoprobe: Au Clusters-Cu^2+^ -Induced Chemiexcitation of Cyclic Peroxides and Resonance Energy Transfer. Chem. Commun..

[128] An H., Guo C., Li D., Liu R., Xu X., Guo J., Ding J., Li J., Chen W., Zhang J. (2020). Hydrogen Peroxide-Activatable Nanoparticles for Luminescence Imaging and In Situ Triggerable Photodynamic Therapy of Cancer. ACS Appl. Mater. Interfaces.

[129] Liu H., Sun M., Su Y., Deng D., Hu J., Lv Y. (2018). Chemiluminescence of Black Phosphorus Quantum Dots Induced by Hypochlorite and Peroxide. Chem. Commun..

[130] Liu H., Su Y., Sun T., Deng D., Lv Y. (2020). Engineering the Energy Gap of Black Phosphorene Quantum Dots by Surface Modification for Efficient Chemiluminescence. Chem. Commun..

[131] Zhang D., Zheng Y., Dou X., Lin H., Shah S.N.A., Lin J.-M. (2017). Heterogeneous Chemiluminescence from Gas–Solid Phase Interactions of Ozone with Alcohols, Phenols, and Saccharides. Langmuir.

[132] Gao H.-Y., Mao L., Li F., Xie L.-N., Huang C.-H., Shao J., Shao B., Kalyanaraman B., Zhu B.-Z. (2017). Mechanism of Intrinsic Chemiluminescence Production from the Degradation of Persistent Chlorinated Phenols by the Fenton System: A Structure–Activity Relationship Study and the Critical Role of Quinoid and Semiquinone Radical Intermediates. Environ. Sci. Technol..

[133] Zou F., Zhou W., Guan W., Lu C., Tang B.Z. (2016). Screening of Photosensitizers by Chemiluminescence Monitoring of Formation Dynamics of Singlet Oxygen during Photodynamic Therapy. Anal. Chem..

[134] Ghogare A.A., Greer A. (2016). Using Singlet Oxygen to Synthesize Natural Products and Drugs. Chem. Rev..

[135] Yang Z., Cao Y., Li J., Lu M., Jiang Z., Hu X. (2016). Smart CuS Nanoparticles as Peroxidase Mimetics for the Design of Novel Label-Free Chemiluminescent Immunoassay. ACS Appl. Mater. Interfaces.

[136] Shu J., Qiu Z., Zhou Q., Lin Y., Lu M., Tang D. (2016). Enzymatic Oxydate-Triggered Self-Illuminated Photoelectrochemical Sensing Platform for Portable Immunoassay Using Digital Multimeter. Anal. Chem..

[137] Zhang H., Zhang X., Dong S. (2015). Enhancement of the Carbon Dots/K_2_S_2_O_8_ Chemiluminescence System Induced by Triethylamine. Anal. Chem..

[138] Liang Y.-F., Wang X., Yuan Y., Liang Y., Li X., Jiao N. (2015). Ligand-Promoted Pd-Catalyzed Oxime Ether Directed C–H Hydroxylation of Arenes. ACS Catal..

[139] Bregnhøj M., Westberg M., Jensen F., Ogilby P.R. (2016). Solvent-Dependent Singlet Oxygen Lifetimes: Temperature Effects Implicate Tunneling and Charge-Transfer Interactions. Phys. Chem. Chem. Phys..

[140] Wilkinson F., Helman W.P., Ross A.B. (1995). Rate Constants for the Decay and Reactions of the Lowest Electronically Excited Singlet State of Molecular Oxygen in Solution. An Expanded and Revised Compilation. J. Phys. Chem. Ref. Data.

[141] Ouyang H., Zhang L., Jiang S., Wang W., Zhu C., Fu Z. (2020). Co Single-Atom Catalysts Boost Chemiluminescence. Chem. Eur. J..

[142] Bhattacharyya A., Dixit A., Banerjee S., Roy B., Kumar A., Karande A.A., Chakravarty A.R. (2016). BODIPY Appended Copper(II) Complexes for Cellular Imaging and Singlet Oxygen Mediated Anticancer Activity in Visible Light. RSC Adv..

[143] McRae E.K.S., Nevonen D.E., McKenna S.A., Nemykin V.N. (2019). Binding and Photodynamic Action of the Cationic Zinc Phthalocyanines with Different Types of DNA toward Understanding of Their Cancer Therapy Activity. J. Inorg. Biochem..

[144] Binder S., Hosikova B., Mala Z., Zarska L., Kolarova H. (2019). Effect of ClAlPcS2 Photodynamic and Sonodynamic Therapy on Hela Cells. Physiol. Res..

[145] Slator C., Molphy Z., McKee V., Long C., Brown T., Kellett A. (2018). Di-Copper Metallodrugs Promote NCI-60 Chemotherapy via Singlet Oxygen and Superoxide Production with Tandem TA/TA and AT/AT Oligonucleotide Discrimination. Nucleic Acids Res..

[146] Kallitsakis M.G., Gioftsidou D.K., Tzani M.A., Angaridis P.A., Terzidis M.A., Lykakis I.N. (2021). Selective C–H Allylic Oxygenation of Cycloalkenes and Terpenoids Photosensitized by [Cu(Xantphos)(Neoc)]BF_4_. J. Org. Chem..

[147] Kallitsakis M.G., Ioannou D.I., Terzidis M.A., Kostakis G.E., Lykakis I.N. (2020). Selective Photoinduced Reduction of Nitroarenes to *N* -Arylhydroxylamines. Org. Lett..

[148] Symeonidis T.S., Athanasoulis A., Ishii R., Uozumi Y., Yamada Y.M.A., Lykakis I.N. (2017). Photocatalytic Aerobic Oxidation of Alkenes into Epoxides or Chlorohydrins Promoted by a Polymer-Supported Decatungstate Catalyst. ChemPhotoChem.

[149] Symeonidis T.S., Tamiolakis I., Armatas G.S., Lykakis I.N. (2015). Green Photocatalytic Organic Transformations by Polyoxometalates vs. Mesoporous TiO _2_ Nanoparticles: Selective Aerobic Oxidation of Alcohols. Photochem. Photobiol. Sci..

[150] Tzani M.A., Terzidis M.A., Lykakis I.N. (2021).

